# The marriage of immunomodulatory, angiogenic, and osteogenic capabilities in a piezoelectric hydrogel tissue engineering scaffold for military medicine

**DOI:** 10.1186/s40779-023-00469-5

**Published:** 2023-07-31

**Authors:** Ping Wu, Lin Shen, Hui-Fan Liu, Xiang-Hui Zou, Juan Zhao, Yu Huang, Yu-Fan Zhu, Zhao-Yu Li, Chao Xu, Li-Hua Luo, Zhi-Qiang Luo, Min-Hao Wu, Lin Cai, Xiao-Kun Li, Zhou-Guang Wang

**Affiliations:** 1 Key Laboratory of Imaging Diagnosis and Minimally Invasive Intervention Research, the Fifth Affiliated Hospital of Wenzhou Medical University, Lishui, 323000 Zhejiang China; 2 Oujiang Laboratory (Zhejiang Lab for Regenerative Medicine, Vision and Brain Health), School of Pharmaceutical Science, Wenzhou Medical University, Wenzhou, 325000 Zhejiang China; 3 Department of Spine Surgery and Musculoskeletal Tumor, Zhongnan Hospital of Wuhan University, Wuhan, 430071 China; 4 National Engineering Research Center for Nanomedicine, College of Life Science and Technology, Huazhong University of Science and Technology, Wuhan, 430074 China; 5 Department of Overseas Education College, Jimei University, Xiamen, 361021 Fujian China; 6 School and Hospital of Stomatology, Wenzhou Medical University, Wenzhou, 325035 Zhejiang China

**Keywords:** Piezoelectric hydrogel, Tissue engineering scaffold, Immunomodulation, Angiogenesis, Osteogenic differentiation

## Abstract

**Background:**

Most bone-related injuries to grassroots troops are caused by training or accidental injuries. To establish preventive measures to reduce all kinds of trauma and improve the combat effectiveness of grassroots troops, it is imperative to develop new strategies and scaffolds to promote bone regeneration.

**Methods:**

In this study, a porous piezoelectric hydrogel bone scaffold was fabricated by incorporating polydopamine (PDA)-modified ceramic hydroxyapatite (PDA-hydroxyapatite, PHA) and PDA-modified barium titanate (PDA-BaTiO_3_, PBT) nanoparticles into a chitosan/gelatin (Cs/Gel) matrix. The physical and chemical properties of the Cs/Gel/PHA scaffold with 0–10 wt% PBT were analyzed. Cell and animal experiments were performed to characterize the immunomodulatory, angiogenic, and osteogenic capabilities of the piezoelectric hydrogel scaffold in vitro and in vivo.

**Results:**

The incorporation of BaTiO_3_ into the scaffold improved its mechanical properties and increased self-generated electricity. Due to their endogenous piezoelectric stimulation and bioactive constituents, the as-prepared Cs/Gel/PHA/PBT hydrogels exhibited cytocompatibility as well as immunomodulatory, angiogenic, and osteogenic capabilities; they not only effectively induced macrophage polarization to M2 phenotype but also promoted the migration, tube formation, and angiogenic differentiation of human umbilical vein endothelial cells (HUVECs) and facilitated the migration, osteo-differentiation, and extracellular matrix (ECM) mineralization of MC3T3-E1 cells. The in vivo evaluations showed that these piezoelectric hydrogels with versatile capabilities significantly facilitated new bone formation in a rat large-sized cranial injury model. The underlying molecular mechanism can be partly attributed to the immunomodulation of the Cs/Gel/PHA/PBT hydrogels as shown via transcriptome sequencing analysis, and the PI3K/Akt signaling axis plays an important role in regulating macrophage M2 polarization.

**Conclusion:**

The piezoelectric Cs/Gel/PHA/PBT hydrogels developed here with favorable immunomodulation, angiogenesis, and osteogenesis functions may be used as a substitute in periosteum injuries, thereby offering the novel strategy of applying piezoelectric stimulation in bone tissue engineering for the enhancement of combat effectiveness in grassroots troops.

**Supplementary Information:**

The online version contains supplementary material available at 10.1186/s40779-023-00469-5.

## Background

In addition to diseases, training injuries and accidental injuries are important causes of non-combat troop reduction in grassroots troops under non-war conditions. Of all injuries, 70.3% were training injuries, and 29.7% were non-training injuries. A total of 66.6% of bone-related injuries were caused by such training and accidental injuries [1, 2]. To establish preventive measures to reduce all kinds of trauma and improve the combat effectiveness of grassroots troops, it is imperative to develop new drugs and scaffolds to promote bone injury repair [3, 4]. Bone injury healing is very challenging due to the risk of uncontrolled and persistent inflammation, disrupted oxygen delivery due to blocked osteogenesis/angiogenesis, and reactive oxygen species (ROS) overload [5]. The use of bone tissue engineering scaffold material, which can provide a microenvironment for bone regeneration, is an effective alternative strategy to support bone regeneration [6]. At present, the effect of many bone tissue engineering scaffolds is close to that of autologous bone transplantation [7], and many new methods, such as electrical stimulation, have been introduced into the field of refractory bone tissue engineering [8]. However, optimizing the combined use of these technologies remains challenging.

Many reports have shown that the electrical microenvironment can play an important role in bone injury repair [9, 10]. In addition, electrical signaling in the body can regulate macrophage behaviors, such as migration, phagocytic activity, and cytokine production [11]. In addition to the incorporation of frequently used bioactive macromolecules and nanomolecules, burgeoning research has found that multiple physiological cues, including mechanics, electricity, and magnetism, represent fresh prospects for bone regeneration by influencing bone-related cell behaviors and cell maturation events [12]. For instance, bone tissue itself is piezoelectric and self-powered in response to body mechanical activities, which can regulate osteocyte metabolism and proliferation [13]. Piezoelectric biomaterials, such as poly-L-lactic acid, collagen, silk, and potassium-sodium niobate, can output the physiological electrical microenvironment and play an important role in augmenting metabolic activities [14, 15]. Importantly, since the discovery of the bioelectric properties of bone 70 years ago, clinical electrostimulation therapy has shown the capability to facilitate bone healing and spinal fusion [16]. Recently, in vitro electrostimulation was shown to have positive effects on the proliferation, migration, and differentiation of bone-forming cells (bone mesenchymal stem cells, osteoprogenitors, osteoblasts, and endothelial cells) [17]. The possible mechanisms by which electrostimulation promotes osteogenesis involve the up-regulation of osteoblast-related intracellular Ca^2+^ concentrations, the primary opening of voltage-gated Ca^2+^ channels, and accelerated osteogenesis through the up-regulation of calmodulin signaling pathways [18].

In addition, the formation of new bone following bone defects is closely related to vascularization, and new blood vessels can participate in a series of physiological and pathological processes of bone regeneration, thereby acting as important links to bone regeneration after bone loss. Excessive oxidative stress decreases the expression of vascular endothelial growth factor (VEGF), greatly hindering the angiogenesis function of the bone defect site. Studies have shown that electrostimulation can promote vascular dilation, enhance vascular permeability, and increase local tissue blood flow [19–21]. Electrical stimulation can also stimulate vascular endothelial cells near the fracture necrosis to neovascularize and grow into the ischemic end, providing blood supply to the injured tissue. Neovascularization is closely related to VEGF expression; however, not all types of electrical stimulation promote VEGF expression [22]. For example, Srirussamee et al. [23] showed that direct current stimulation did not promote the expression of VEGF in preosteoblasts. Kim et al. [24] confirmed that alternating current (AC) stimulation could significantly up-regulate VEGF expression when measured by RT-PCR and ELISA experiments. In our previous research, we constructed piezoelectric films by combining piezoelectric nanomaterials, which could generate AC driven by mechanical forces [25, 26]. In addition, we demonstrated that AC stimulation could effectively promote the expression of VEGF in tissue defect sites and thus boost the generation of new blood vessels [19]. Dobashi et al. [27] developed piezoionic hydrogels that could transduce pressure stimuli into currents for neuromodulation applications. Given this context, there is an urgent need to develop a bone tissue engineering scaffold material that can both regulate the immune microenvironment and generate AC under mechanical action.

In this study, a piezoelectric hydrogel bone scaffold was fabricated by doping polydopamine (PDA)-modified barium titanate (PDA-BaTiO_3_, PBT) nanoparticles and polydopamine-modified ceramic hydroxyapatite (PDA-HA, PHA) nanoparticles into chitosan/gelatin [Cs/Gel (CG)] hydrogels. The physical and chemical properties were tested to verify that the change in the properties of the piezoelectric hydrogel was due to the introduction of BaTiO_3_. As the Cs/Gel/PDA-modified HA/PDA-modified BaTiO_3_ (CG/PHA/PBT) piezoelectric hydrogels could provide in situ stabilized electrostimulation, the utility of the CG/PHA/PBT piezoelectric hydrogels for large-sized bone repair was assessed to exam the in vivo pro-healing ability, immunomodulatory function, and angiogenic and osteogenic activity. Finally, RNA transcriptomics and inhibition experiment analysis was conducted to verify the key signaling pathway to regulate bone regeneration by piezoelectric hydrogels.

## Methods

### Materials

Cs, Gel, and dopamine hydrochloride were purchased from Sigma-Aldrich Co., Ltd. (9012-76-4, 9000-70-8, H8502; St. Louis, MO, USA). BaTiO_3_ nanoparticles were provided by Alfa Aesar (12047-27-7; Wardhill, MA, USA). For cell culture experiments, fetal bovine serum, alpha-modified Eagle’s medium (α-MEM), Dulbecco’s modified Eagle’s medium (DMEM), phosphate-buffered saline (PBS), trypsin-ethylene diamine tetraacetic acid (EDTA), and penicillin/streptomycin (P/S) were sourced from Gibco Life Technologies Co. (10100147, 12571063, 11965092, 70011044, 17892, 15070063; Grand Island, USA). The cell counting kit-8 (CCK-8) was purchased from Dojindo Laboratories (CK04; Kumamoto, Japan). The live/dead cell staining kit was obtained from BestBio Biotechnologies (C2015M; Shanghai, China). Triton X-100 (9036-19-5; Sigma-Aldrich), DAPI (28718-90-3; Sigma-Aldrich), and TRITC-labeled phalloidin (A34055; Invitrogen, USA) were used for cell staining. The Hoechst 33342 stain, BeyoClick™ EdU-555 imaging kit, Annexin V-FITC apoptosis detection kit, TRIzol RNA extraction kit, radioimmunoprecipitation assay (RIPA) lysis buffer, 5-bromo-4-chloro-3-indolyl phosphate/nitro blue tetrazolium (BCIP/NBT), alkaline phosphatase (ALP) color development kit, and bicinchoninic acid (BCA) protein assay kit were supplied by Beyotime Biotechnology Co., Ltd. (C1022, C0075S, C1062S, R0016, P0013B, C3206, P0321M, P0009; Jiangsu, China). The alizarin red S (ARS) staining solution was acquired from Servicebio Co., Ltd. (G1038; Wuhan, China). All chemicals were used without further purification. The water used in all experiments was purified by a Milli-Q cycle purification system (Millipore, USA).

### Fabrication of piezoelectric hydrogels

**Fig. 1:**
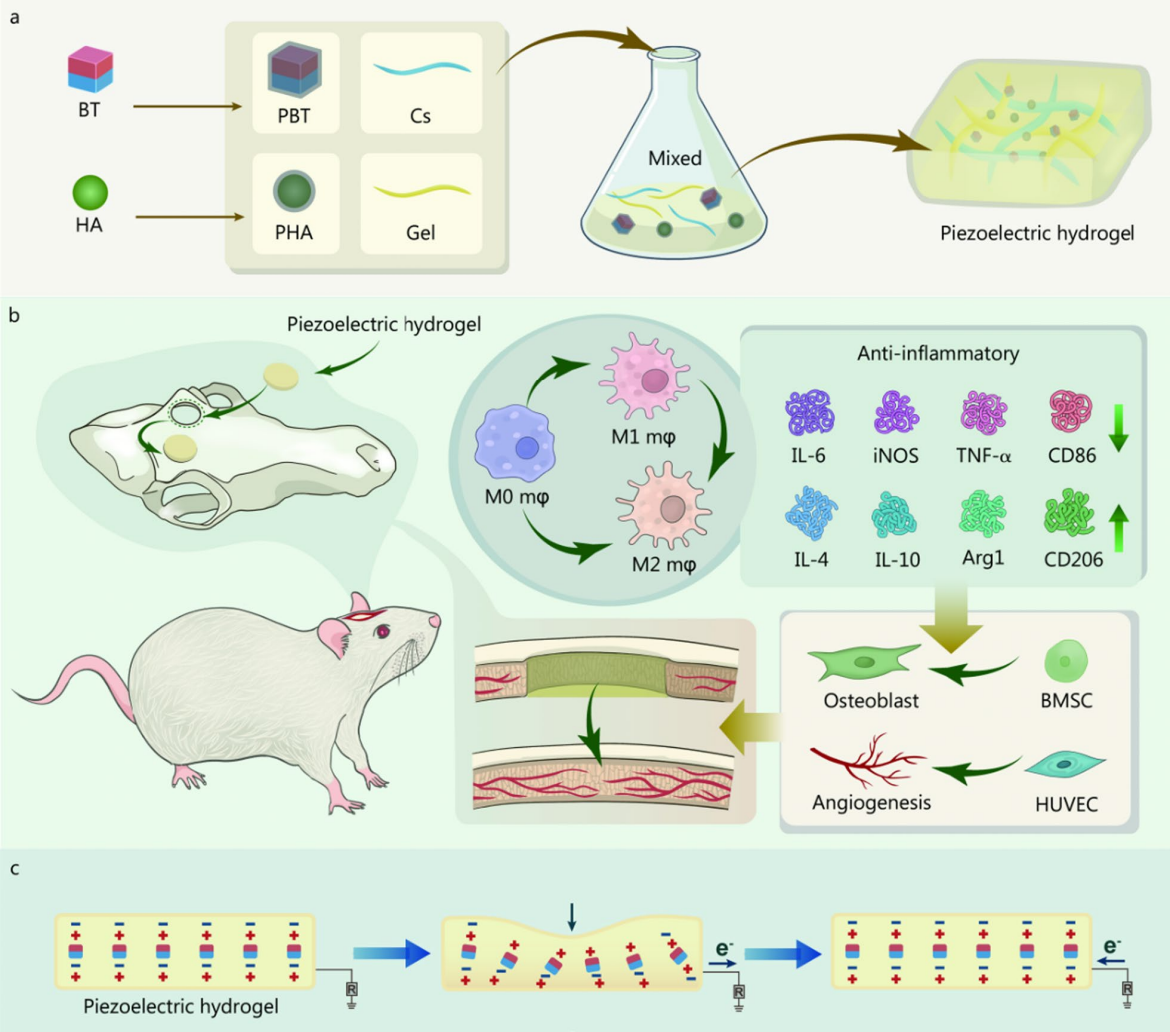
Schematic illustration of the design strategy of the piezoelectric CG/PHA/PBT hydrogels and the bone regeneration mechanism. **a** The preparation process of the piezoelectric CG/PHA/PBT hydrogels. **b** The application and potential biological mechanism of the piezoelectric hydrogels for rapid bone regeneration. **c** Schematic illustration of the possible self-powered mechanism of the piezoelectric CG/PHA/PBT hydrogels. The dipoles in the BT nanoparticles are oriented in the same direction in the piezoelectric hydrogels. The electric polarization is presented in the direction of the oriented dipoles and can produce a piezoelectric potential in the BT piezoelectric nanoparticles. In the absence of external stimuli, there is a positive and negative charge balance in the hydrogel. Once a pressure stimulus is applied to the piezoelectric hydrogels, the electrons in the hydrogels flow out of the hydrogel. Once the pressure disappears, the cumulative free charges flow back into the piezoelectric hydrogels. BT barium titanate, HA hydroxyapatite, Cs chitosan, Gel gelatin, PHA polydopamine coated-hydroxyapatite, PBT polydopamine coated-barium titanate, e^−^ electron, R resistance, M0 mφ unpolarized macrophage, M1 mφ type 1 macrophage, M2 mφ type 2 macrophage, IL-6 interleukin-6, IL-4 interleukin-4, TNF-α tumor necrosis factor-α, iNOS inducible nitric-oxide synthase, CD86 cluster of differentiation 86 protein, Arg1 arginase, BMSC bone marrow stromal cell, HUVEC human umbilical vein endothelial cell

The preparation of PHA and PBT is shown in Fig. 1a, 1 g of HA or BaTiO_3_ nanoparticles was dispersed into a PDA solution and kept at 37 °C for 12 h. The nanoparticles were collected by gradient centrifugation. For the preparation of the piezoelectric hydrogels, Cs was dissolved in 1% acetum [2% (w/v)], and Gel was dissolved completely in deionized water [2% (w/v)]. The Cs and Gel solutions were mixed in a 1:1 ratio to form a CG solution. The PHA powder was added to the CG solution at 10% of the total weight of the CG to obtain a CG/PHA suspension. Then, the PBT powder was added to the above solution at 0, 5, and 10 wt% ratios of the total weight. One hundred microliters of genipin solution [2% (w/v)] was serially added to 10 ml aliquots of the CG/PHA/PBT solution, stirred for 10 min until blended, and then poured into plates for gel formation. In the following sections, pure CG hydrogels are denoted as the CG group, nanocomposite CG hydrogels with PHA functionalization are denoted as the CG/PHA group, and nanocomposite CG hydrogels with 5 wt% and 10 wt% PBT functionalization are denoted as the CG/PHA/5%PBT and CG/PHA/10%PBT groups, respectively.

### Characterization of the piezoelectric hydrogels

After freeze-drying the piezoelectric hydrogels, the topography of the piezoelectric hydrogels was characterized with field-emission scanning electron microscope (FE-SEM; Zeiss, Sigma, Germany) and the Ca, P, Ba and Ti element distribution in the piezoelectric hydrogels was scanned with the energy dispersive spectrometer (UltimMax 40, Oxford, UK). The function group changes in the piezoelectric hydrogels were tested by X-ray diffraction (XRD; Rigaku, Japan) and fourier transform infrared spectroscopy (FTIR; TNZ1-5700, Nicolet, USA). The elasticity modulus of piezoelectric hydrogels was conducted with a rheometer (Kinexus, Malvern). The output voltages of the piezoelectric hydrogels were determined by a digital storage oscilloscope (RTM3000, Rohde & Schwarz, Germany).

### In vitro biocompatibility evaluation

The RAW 264.7 cells (murine-derived macrophage cell line) were provided by the Institute of Biochemistry and Cell Biology of the Chinese Academy of Sciences (Shanghai, China) (ab275474; Abcam, USA). The CCK-8 assay was performed to evaluate the cytotoxicity of the piezoelectric hydrogels. The extracts from the piezoelectric hydrogels were obtained according to ISO1099312:2007. The culture medium without the extracts served as the control. After 1, 2, and 3 d of culture, the CCK-8 solution was added to each well (20 μl per well) and the cells were incubated for another 4 h at 37 °C with 5% CO_2_. Besides, the live/dead staining was conducted to verify the viability of RAW 264.7 cells on different piezoelectric hydrogels. The live cells were stained green by Calcein-AM and the dead cells were stained red by propyl iodide (PI). The 3D culture of macrophages (RAW 264.7 cells) with the piezoelectric hydrogels was conducted similar to the 2D culture. To construct 3D images, the original data acquired through Z-stacking were processed using Leica black/blue software (Leica). All of these experiments to evaluate the cell apoptosis were conducted on the piezoelectric hydrogels.

### In vitro immunomodulation of macrophages

First, immunofluorescence was used to observe the morphological changes in macrophages cocultured on different samples. Briefly, after coculturing for 2 d, the samples were stained with F-actin and F4/80, and photographs were taken using a confocal microscope. Next, for phenotypic polarization, surface markers of M1 macrophages (CD86) and M2 macrophages (CD206) were detected using flow cytometry as previously described [28]. RAW 264.7 cells were first treated with lipopolysaccharide (LPS) for 24 h as a simple in vitro stimulus of the inflammatory microenvironment during bone healing. Harvested cells were then seeded onto each sample in a 24-well tissue culture plate at a density of 3 × 10^4^ cells per well. After incubation for 2 d, the cells were collected by trypsinization and then blocked with 1% bovine serum albumin (BSA) for 20 min. After rinsing twice with PBS, allophycocyanin (APC)-conjugated CD86 (105011, BioLegend, China) and FITC-conjugated CD206 (141703, BioLegend, China) were used for surface antigen staining for 30 min. The treated cells were analyzed by flow cytometry (FC500, USA) using FlowJo software (Tree Star Inc., USA). The polarization of macrophages was analyzed by immunofluorescence staining and Western blotting assays. For immunofluorescence staining, a mouse monoclonal antibody against inducible nitric-oxide synthase (iNOS; ab210823, Abcam, UK) and CD206 (M2 marker, PA5-101657, Invitrogen, USA) antibodies were used to detect the polarization of macrophages. The primary antibodies against iNOS (GTX130246, Genetex, USA), CD206 (24595S, Cell Signaling, USA), and GAPDH (ab8245, Abcam, UK) were incubated for Western blotting assay.

The anti-inflammatory efficacy of the piezoelectric hydrogel was further analyzed by RT-qPCR. Briefly, after coculturing for 2 d, total RNA isolation, complementary DNA (cDNA) synthesis, and RT-qPCR were performed as described above. Gene expression levels were normalized to those of the endogenous housekeeping gene *GAPDH*. The primer sequences of each gene are presented in Additional file 1: Table S1.

After the macrophages were cocultured for 2 d, the supernatant of each group was collected for further study based on previously reported protocols [29]. After centrifugation at 8000 r/min for 5 min and filtration through a 0.22-μm filter, the sterile supernatants were collected carefully and stored at 4 °C. In addition, the concentrations of BMP-2, TGF-β1, VEGF, and bFGF secreted by cells in the collected supernatant were measured with corresponding ELISA kits. All ELISA measurements were conducted according to the manufacturer’s instructions.

RNA-seq was carried out to study the gene expression profiles of macrophages cultured on different hydrogels. Briefly, RAW 264.7 macrophages (1 × 10^6^ cells/ml) were co-cultured on different hydrogel samples in 24-well plates for 2 d. Then, total RNA from the macrophages of each group was collected using TRIzol reagent following the manufacturer’s instructions. RNA samples were stored at –80 °C before sequencing. Finally, RNA sequencing was performed using BMK Cloud (https://www.biocloud.net/). The expression of several selected genes was examined using a heatmap and evaluated by Kyoto Encyclopedia of Genes and Genomes (KEGG) pathway analysis.

### Inhibition experiments

To verify the signaling pathways possibly involved in the contribution of the piezoelectric hydrogels to M2 polarization, an inhibitor (BEZ235, 0–200 nmol/L, Selleck, USA) of PI3K was added in complete cell culture medium to culture RAW 264.7 cells on piezoelectric hydrogels. LPS (200 ng/ml) was co-cultured with macrophages as the negative control. Interleukin-4 (IL-4, 20 ng/ml) was also co-cultured with macrophages as positive control. After 48 h, the RAW 264.7 cells were harvested for an immunofluorescence staining assay to detect their polarization.

### In vitro evaluation of angiogenesis

Human umbilical vein endothelial cell (HUVEC; CRL-1730, ATCC, USA) migration activity promoted by the piezoelectric hydrogels was determined by Transwell and in vitro scratch wound assays [30]. For the Transwell chamber assay, the HUVEC cells were seeded in the upper chamber. The macrophage medium was added in the lower chamber. The migrated cells on the lower surface were stained with 0.1% crystal violet solution for observation. For the scratch wound assay, when the HUVECs reached 90% confluence, a straight scratch was created. The macrophage media was added for stimulating the HUVEC migration. The HUVECs cells were fixed and then stained with crystal violet solution for migration observation.

The vessels formation assay was measured with HUVECs under different macrophage media to induce the angiogenesis. In brief, the HUVEC cells were seeded in the growth factor reduced basement membrane matrix (Matrigel, 356231, Corning, USA) and incubated with different hydrogel extracts. Then the tube-like structures were observed and quantitatively analyzed. Besides, the expression of VEGF, hypoxia inducible factor-1α (HIF-1α), bFGF, and angiopoietin-1 (Ang-1) was measured by RT-qPCR. All primer sequences used in RT-qPCR are presented in Additional file 1: Table S1. After culturing for 7 d, immunofluorescence staining was performed to detect neovascularization as described before [29].

### In vitro osteogenic activity of MC3T3-E1

MC3T3-E1 (CRL-2593, ATCC, USA) migration activity was determined by Transwell and in vitro scratch wound assays. The experimental procedures were the same as above. MC3T3-E1 cells (5 × 10^4^ cells per well) were co-cultured with conditioned macrophage medium and complete medium (1:1), in which included 10 mmol/L β-glycerophosphate disodium salt, 50 μg/ml ascorbic acid, and 10 nmol/L dexamethasone. ALP staining was conducted with BCIP/NBT staining kit according to the manufacturer’s protocol. On day 14, the fixed cell samples were immersion in ARS solution for ARS staining. The cells were co-incubated with 10% cetylpyridinium chloride for 30 min with shaking. After centrifugation for 15 min, the collected supernatant of each sample was measured at a wavelength of 562 nm. After coculturing for 7 d, the cells were fixed and incubated with 5% goat serum. Then the samples were incubated with primary target antibodies against runt-related transcription factor 2 (Runx2; D1L7F, Cell Signaling) and osteopontin (OPN; 22952–1-AP, ProteinTech). After incubation with the fluorescence-labeled secondary antibody, and being counterstained with DAPI, they were observed with CLSM (TCS SP8, Leica, Germany).

The expression levels of Runx2, collagen type I (Col-1), OPN, and osteocalcin (OCN), in MC3T3-E1 cells were examined with qRT-PCR. Briefly, total RNA was isolated from the cells and cDNA was synthesized using a HiScript III RT SuperMix reverse transcription kit. The forward and reverse primers for the selected genes are also listed in Additional file 1: Table S1.

### In vivo bone defect repair capacity

The animal experiments were approved by the Animal Care and Use Committee of Zhongnan Hospital of Wuhan University (No. ZN2022282). As shown in Fig. 1b, a skull bone defect injury animal model was constructed. Thirty-six male rats (200–220 g) were randomly divided into four groups: control, CG, CG/PHA, and CG/PHA/5%PBT. Animal experiments flow chart and corresponding tests are shown in Additional file 2: Fig. S1. Two weeks post-surgery, 3 rats in each group were sacrificed using excess pentobarbital sodium, and half of the calvarial bone was harvested and fixed in 4% paraformaldehyde for 48 h at 4 °C for further analysis. The samples were then decalcified by soaking them in 10% EDTA for approximately 4 weeks at 37 °C. After embedding them in paraffin, the bone defect area containing membranes was cut to prepare paraffin sections with a thickness of 5 μm. To evaluate the local inflammatory response at the initial stage after implantation, iNOS and CD206 were selected as specific markers for double-labeled immunofluorescence staining to observe the phenotype of the infiltrating macrophages. Furthermore, immunohistochemical staining for the pro-inflammatory factor TNF-α and the anti-inflammatory cytokine IL-10 was performed to assess the anti-inflammatory properties of the piezoelectric hydrogel during bone healing. Immunohistochemical and immunofluorescence staining for the pro-angiogenesis factor VEGF/CD31 and the pro-osteogenic differentiation cytokine bone morphogenetic protein 2 (BMP-2)/Runx2 were further performed to assess the vascularization and osteogenic differentiation of the samples by piezoelectric hydrogel treatment during bone healing.

Eight weeks post-surgery, 3 rats in each group were sacrificed as mentioned above, and the whole calvarial bone was scanned by a micro-CT system (SkyScan 1276 system, Bruker, Germany) with the following settings: voltage, 200 kV; current, 65 μA; and filtration, 0.25 mm aluminum with an image pixel size of 6.5 μm. The bone tissue volume/total tissue volume (BV/TV) and bone mineral density (BMD) were analyzed based on the reconstructed micro-CT images. The last 3 rats were killed and the samples were harvested for histological analysis. The tissue samples were sectioned and stained with hematoxylin and eosin (HE) to evaluate bone repair capability. Afterward, Masson’s trichrome staining and Goldner’s trichrome staining were performed to visualize the maturation of regenerated bone tissue in the defect area. For Masson’s trichrome staining, the red areas were regarded as mineralized collagen (mature bone matrix), while the blue areas were regarded as non-mineralized collagen (immature bone matrix). For Goldner’s trichrome staining, the orange/red-stained areas were regarded as osteoid (immature bone), while the dark-green-stained areas were regarded as mineralized bone (mature bone). Additionally, in vivo osteogenesis and vascularization in the bone defects were analyzed by the immunohistochemical staining of Col-1, Runx2, OPN, CD31, and CD34 as previously described [32]. All stained samples were imaged with microscope and semi-quantified by ImageJ software.

### Statistical analysis

All experiments in this work were performed at least three times. All data are presented as the mean ± standard deviation (SD), and graphs were fabricated with Origin 2018 software (Origin Lab Corporation, USA). Analyses were performed with Student’s *t*-test (unpaired and two-tailed), one-way or two-way ANOVA, followed by Tukey post hoc. *P*-value < 0.05 was considered statistically significant.

## Results

### Characterization of the piezoelectric hydrogels

**Fig. 2:**
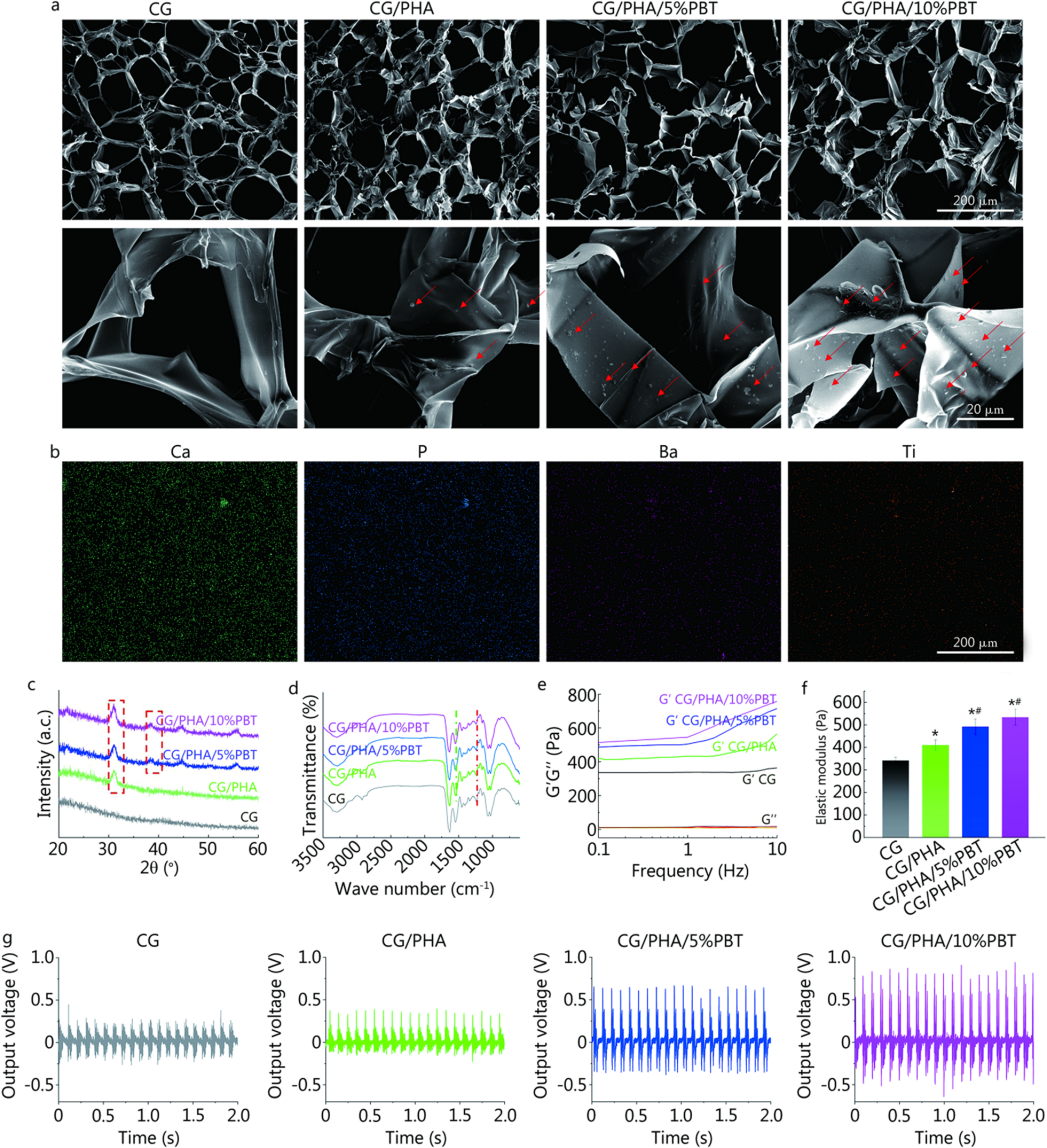
Characterization of the piezoelectric hydrogels. **a** Representative SEM images of different hydrogel samples, the red arrows represent piezoelectric nanoparticles. **b** EDS elemental mapping of the CG/PHA/5%PBT piezoelectric hydrogel. XRD (**c**), FTIR (**d**), rheological curve (**e**), and elasticity modulus (**f**) of different hydrogel samples. **g** The output voltage of different hydrogel samples under 10 Hz and 1 kP pressure. ^*^P < 0.05, compared with the CG group; ^#^*P* < 0.05, compared with the CG/PHA group. CG chitosan/gelatin, PHA polydopamine coated-hydroxyapatite, PBT polydopamine coated-barium titanate, SEM scanning electron microscope, EDS energy disperse spectroscopy, XRD X-ray diffraction, FTIR fourier transform infrared spectroscopy

As shown in Figs. 1a and 2a, a porous piezoelectric hydrogel scaffold was fabricated by doping PDA-modified ceramic HA and piezoelectric BaTiO_3_ nanoparticles into CG hydrogels. The CG hydrogel containing 10% w/w PDA-modified HA nanoparticles was named CG/PHA. The CG hydrogels containing 10% w/w PDA-modified HA nanoparticles and 5% w/w or 10% w/w PDA-modified BaTiO_3_ nanoparticles were named CG/PHA/5%PBT and CG/PHA/10%PBT, respectively. The CG hydrogel presented a smooth surface, and the surface of the hydrogels with incorporated HA and piezoelectric BaTiO_3_ nanoparticles showed the presence of nanoparticles. The surface roughness of the hydrogels increased with an increase in the number of nanoparticles, but the porosity of the hydrogel did not change. To solve the problem of nanoparticle dispersion, the HA and piezoelectric BaTiO_3_ nanoparticles were modified with PDA. A layer coating the surface of the BaTiO_3_ nanoparticles could be clearly seen through transmission electron microscopy (TEM) (Additional file 2: Fig. S2). According to Fig. 2b, energy disperses spectroscopy (EDS) mapping of the CG/PHA/5%PBT sample doped with HA nanoparticles (10% w/w) and piezoelectric BaTiO_3_ nanoparticles (5% w/w) showed that the special elements (Ca and P present in HA; Ba and Ti present in BaTiO_3_) were uniformly distributed, indicating that the HA and piezoelectric BaTiO_3_ nanoparticles were evenly distributed in the hydrogel without focusing. Due to the introduction of HA nanoparticles into the hydrogels, a characteristic peak at 2θ = 31.7° could be observed (Fig. 2c). The characteristic peak was assigned to the (211) reflection planes and was consistent with the reported HA phase (JCPDS Card No. 01–74-0566). Moreover, for the diffraction curve of PHA/PBT, the splitting peaks at approximately 45.4° belonged to the (002) planes for the tetragonal phase of BaTiO3 (JCPDS Card No. 05–0626), which proved its ferroelectric/piezoelectric properties [33]. Furthermore, the intensity of the peaks increased with increasing PBT content in the hydrogels. The FTIR spectra of the piezoelectric hydrogels are displayed in Fig. 2d. It was clear that the band at 1272 cm^−1^ was assigned to C–O–C stretching, which could be ascribed to the Cs and Gel components. Compared with the CG hydrogel, the absorption band located at 1510 cm^−1^ corresponded to the shear vibration of N–H (Fig. 2d), implying the presence of PDA [34].

The storage modulus of CG, CG/PHA, CG/PHA/5%PBT and CG/PHA/10%PBT piezoelectric hydrogels was 342.8, 408.4, 493.1 and 532.2 Pa, respectively (Fig. 2e, f). With the incremental weight ratio of HA and BaTiO_3_ nanoparticles, the storage modulus (G′) of the CG/PHA/PBT piezoelectric hydrogels increased (*P* < 0.05), which may be caused by the up-regulation of cross-linking density in the CG/PHA/PBT hydrogels. To detect the electricity generation performance of the CG/PHA/PBT piezoelectric hydrogels, a voltage output test was performed. All the composite hydrogels showed a certain generating capacity (Fig. 2g), and the voltage output of the CG, CG/PHA, CG/PHA/5%PBT, and CG/PHA/10%PBT hydrogels was approximately 0.2, 0.25, 0.6, and 0.8 V, respectively. The electricity generation characteristics of CG/PHA hydrogels with 5–10 wt% BaTiO_3_ reflected an increased ability to self-generate electricity. As demonstrated in Fig. 1c, the possible self-powered mechanism underlying the piezoelectric signal in the CG/PHA/PBT hydrogels can be described as follows. The dipoles in the BaTiO_3_ nanoparticles are oriented in the same direction in the CG/PHA/PBT piezoelectric hydrogels. The electric polarization is presented in the direction of the oriented dipoles and can produce a piezoelectric potential in the BaTiO_3_ piezoelectric nanoparticles. In the absence of external stimuli, there is a positive and negative charge balance in the hydrogel. Once a pressure/strain stimulus is applied to the piezoelectric hydrogels, the electrons in the hydrogels flow out of the hydrogel. Once the pressure/strain disappears, the cumulative free charges flow back into the piezoelectric hydrogels. During this series of actions, a positive and a negative current can be observed. If a force stimulus is continuously applied and released (allowing the gel to return to its original volume), a typical sinusoidal electric signal will be observed, as illustrated in Fig. 2g.

These results indicate the successful synthesis of a hydrogel scaffold material with piezoelectric properties. This scaffold material can provide effective electrical stimulation under external pressure and form a good electrical microenvironment for tissue regeneration.

### Immunomodulation based on the piezoelectric hydrogels

**Fig. 3:**
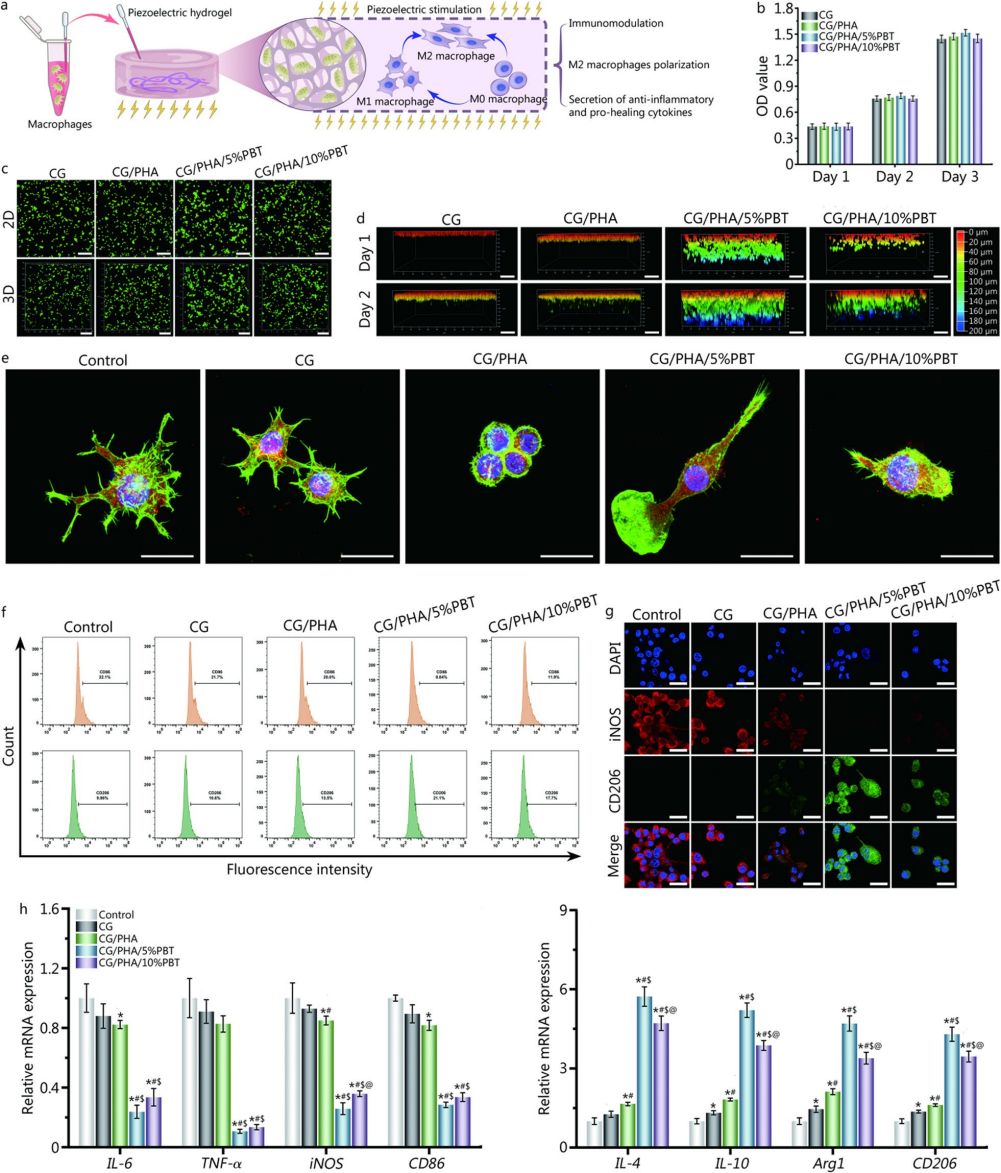
In vitro evaluation of the immunomodulatory properties of the piezoelectric hydrogels. **a** Schematic diagram of immune regulation by the piezoelectric hydrogels. **b** Evaluation of the cytocompatibility of the piezoelectric hydrogels. **c–d** 2D and 3D live and dead cell staining of macrophages (RAW 264.7 cells) cocultured with the piezoelectric hydrogels. Scale bar: 200 μm (**c**), 100 μm (**d**). **e** Representative immunofluorescence images of RAW 264.7 cells on hydrogels. F-actin (green) is a fibrous actin that presents the skeleton of the cells, F4/80 (red) is cell surface glycoprotein and the marker of mature mouse macrophages, DAPI (blue) stands for the nucleus. Scale bar: 25 μm. **f** Representative flow cytometric dot plots showing cell surface markers of RAW 264.7 cells, including CD86 and CD206. **g** Representative immunofluorescence images of iNOS (red) and CD206 (green) in RAW 264.7 cells on hydrogels on day 2. Scale bar: 25 μm. **h** Relative mRNA expression levels of anti-inflammatory genes and pro-inflammatory genes of macrophage under the piezoelectric hydrogels stimulation for 2 d. ^*^P < 0.05, compared with the control group; ^#^*P* < 0.05, compared with the CG group; ^$^*P* < 0.05, compared with the CG/PHA group; ^@^*P* < 0.05, compared with the CG/PHA/5%PBT group. CG chitosan/gelatin, PHA polydopamine coated-hydroxyapatite, PBT polydopamine coated-barium titanate, IL-6 interleukin-6, TNF-α tumor necrosis factor-α, iNOS inducible nitric-oxide synthase, IL-4 interleukin-4, IL-10 interleukin-10, Arg1 arginase 1

During the bone healing process, inflammation, oxidative stress, angiogenesis, and osteogenesis are closely connected. The first-stage immunoreaction after scaffold embedment is extremely important for bone tissue repair. Overlong immunoreactions during the bone-repairing process delay bone regeneration, eventually leading to the non-union. Moreover, the transformation of macrophage phenotypes during inflammation affects the subsequent cascade of events, which determines the fate of implanted scaffolds. Figure 3a shows how to coculture the piezoelectric hydrogels with macrophages and depict the phenotypic changes and immunomodulatory effects of macrophages under the influence of the piezoelectric hydrogels. Before the cell experiments were performed, the cytocompatibility of the piezoelectric hydrogels with RAW 264.7 cells was determined with the CCK-8 kit test. As seen in Fig. 3b, no piezoelectric hydrogel extract groups showed toxic effects on RAW 264.7 cells at different time points. The cell viability of the piezoelectric hydrogel extract groups was similar to that of the untreated group (*P* > 0.05). To further confirm the CCK-8 results, cell apoptosis was analyzed by live/dead cell staining (Fig. 3c, d). Both two-dimensional (2D) and three-dimensional (3D) images of RAW 264.7 cells on the piezoelectric hydrogels showed a high survival rate and no apoptotic cells. Moreover, the migration behavior of macrophages could be observed in 3D culture. Among the four groups of hydrogel scaffold materials, the RAW 264.7 cells in the CG/PHA/5%PBT group migrated most deeply to the hydrogel void.

The immunomodulatory properties of the piezoelectric hydrogels were studied. Figure 3e shows the immunofluorescence staining of F-actin and F4/80 in RAW 264.7 cells on piezoelectric hydrogel scaffolds. The F-actin and F4/80 double-labeled immunofluorescence images showed that macrophages adhered to the surface of the CG hydrogel, and the cells in the control group presented a fried egg-like shape with many filopodia, while those in the CG/PHA group had a typical round appearance. Additionally, the macrophages adhering to the surface of the CG/PHA/5%PBT and CG/PHA/10%PBT groups adopted a spindle-like shape. The cell spreading area and spindle cell ratio results showed that macrophages in the CG and control groups presented an M1-like morphology and large spreading area, while the macrophages in the CG/PHA/5%PBT and CG/PHA/10%PBT groups exhibited a spindle-like shape, which is considered typical of the M2 phenotype. Therefore, the immunomodulatory effects of the piezoelectric hydrogels caused more proneness to M2-type macrophage activation in the piezoelectric hydrogel groups than in the non-piezoelectric hydrogel groups.

In addition, flow cytometry was used to evaluate macrophage polarization. The CD86 positive cell ratios of control, CG, CG/PHA, CG/PHA/5%PBT and CG/PHA/10%PBT groups were 21.7%, 21.4%, 20.0%, 8.8% and 11.9%, respectively. The CD206 positive cell ratios of control, CG, CG/PHA, CG/PHA/5%PBT and CG/PHA/10%PBT groups were 9.9%, 10.6%, 13.5%, 21.1% and 17.7%, respectively (Fig. 3f, Additional file 2: Fig. S3a). Flow cytometric staining and quantitative results showed that the piezoelectric hydrogels significantly reduced CD86 (an M1 macrophage surface marker) expression and enhanced CD206 (an M2 macrophage surface marker) expression (*P* < 0.05). Higher expression of CD86 was observed in the control, CG and CG/PHA groups than in macrophages exposed to the CG/PHA/5%PBT and CG/PHA/10%PBT groups. Meanwhile, the number of CD206^+^ M2 macrophages was increased in the CG/PHA/5%PBT and CG/PHA/10%PBT groups compared to the control, CG and CG/PHA groups (*P* < 0.05; Additional file 2: Fig. S3a). The immunofluorescence and Western blotting results were similar to those of flow cytometry (Fig. 3g, Additional file 2: Fig. S3b).

To investigate the cytokine profile, RT-qPCR experiments were performed (Fig. 3h). The RT-qPCR results showed the significant down-regulation of pro-inflammatory cytokines, such as *IL-6*, *TNF-α*, *iNOS*, and *CD86*, in the CG/PHA/5%PBT and CG/PHA/10%PBT groups (*P* < 0.05). Meanwhile, the expression of anti-inflammatory gene markers (*IL-4*, *IL-10*, *Arg1*, and *CD206*) was significantly up-regulated in these groups compared to the CG/PHA, CG, and control groups (*P* < 0.05). Moreover, the secretion of BMP-2, TGF-β1, VEGF, and bFGF illustrated that the electrical microenvironment elevated the accommodation effects in accelerating pro-regeneration cytokine secretion by macrophages (*P* < 0.05, Additional file 2: Fig. S4).

The above results suggest that the electrical cues and electrical microenvironment provided by the CG/PHA/PBT piezoelectric hydrogels could regulate immunity and promote M2 macrophage polarization and anti-inflammatory and pro-healing cytokine secretion by macrophages. Since the biological performance of the CG/PHA/5%PBT hydrogel was better than that of the CG/PHA/10%PBT piezoelectric hydrogel, we focused on the CG/PHA/5%PBT hydrogel in subsequent experiments.

### Effects of the piezoelectric hydrogels on angiogenesis

**Fig. 4:**
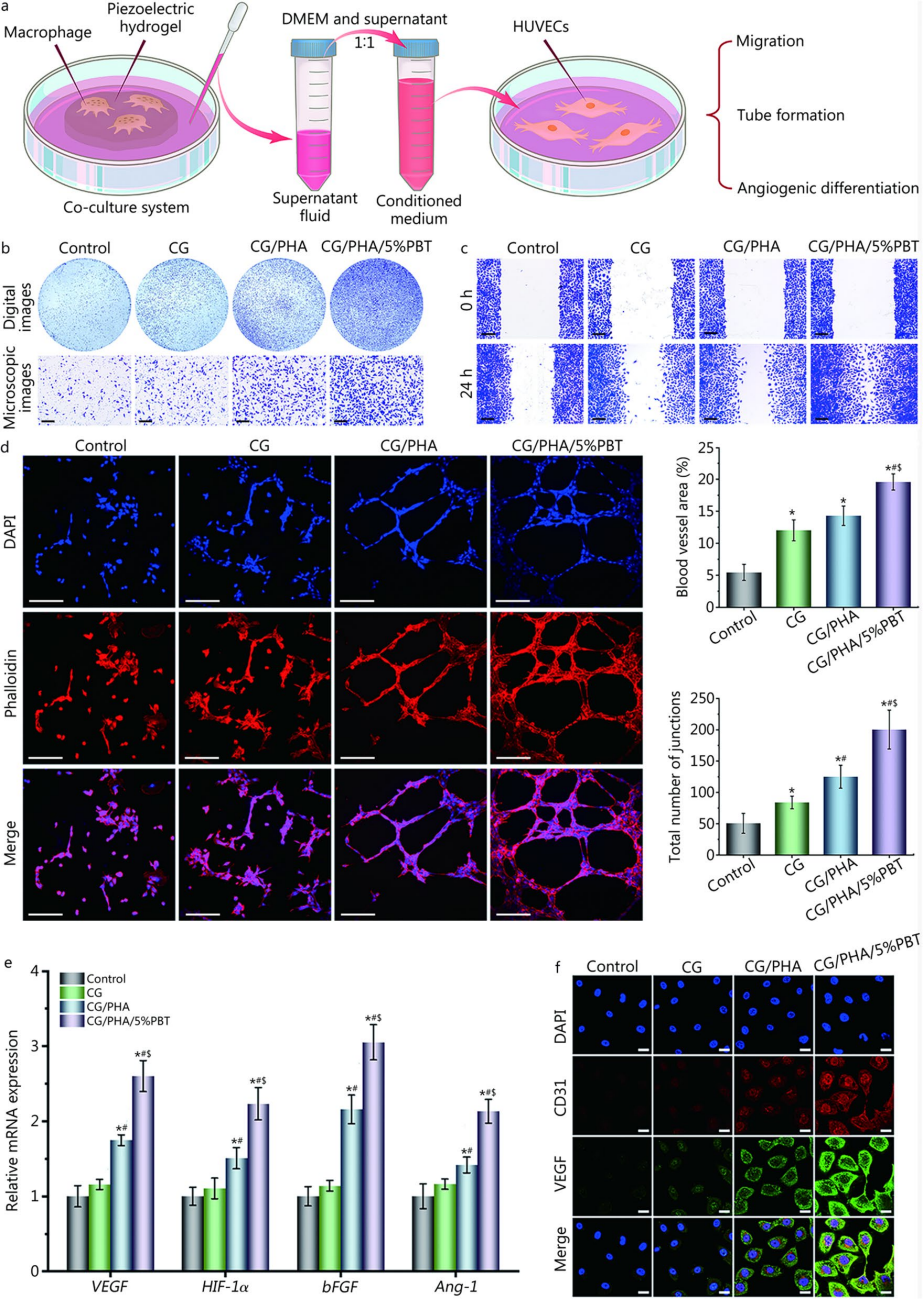
In vitro assessment of the angiogenic ability of the piezoelectric hydrogels. **a** Schematic diagram of the pro-angiogenic cell experiment. First, the piezoelectric hydrogel was co-cultured with macrophages; then, vascular endothelial cells were cultured using the co-culture medium from macrophages.** b** Representative digital and microscopic images of the migrated HUVECs cells to the lower chamber after the macrophage medium was separately added to the lower chamber and cultured for 24 h. Scale bar: 200 μm. **c** Representative microscopic images of HUVECs after the cells were co-cultured with different macrophage media for 24 h. Scale bar: 200 μm. **d** Representative fluorescence images and quantitative analysis of HUVEC tube formation after the cells were co-cultured with different macrophage media for 8 h. Percentage of blood vessel area is the vessel density of vascular endothelial cells in a single image, total number of junctions is the cell intersections between vascular endothelial cells in a single image, tube formation was quantified using AngioTool (National Cancer Institute, NIH) for the percentage of blood vessel area and the total number of junctions. Scale bar: 250 μm. **e** Relative mRNA expression levels of angiogenesis-related genes in HUVECs after the cells were co-cultured with different macrophage media for 7 d, including *VEGF*, *HIF-1α*, *bFGF*, and *Ang-1*. **f** Representative immunofluorescence images of CD31 (red), VEGF (green), and nuclei (blue) in HUVECs after the cells were co-cultured with different macrophage media for 7 d. Scale bar: 25 μm. ^*^P < 0.05, compared with the control group; ^#^*P* < 0.05, compared with the CG group; ^$^ *P* < 0.05, compared with the CG/PHA group. CG chitosan/gelatin, PHA polydopamine coated-hydroxyapatite, PBT polydopamine coated-barium titanate, VEGF vascular endothelial growth factor, HIF-1α hypoxia inducible factor-1α, bFGF basic fibroblast growth factor, Ang-1 angiopoietin-1, HUVECs human umbilical vein endothelial cells

To study the chemotactic response of HUVECs to the co-culture medium from the co-culture of macrophages and the piezoelectric hydrogels, Transwell migration and scratch wound healing assays were performed. As shown in Fig. 4a-c and Additional file 2: Fig. S5, the co-culture medium promoted the migration and infiltration of HUVECs. These behavioral changes are very beneficial to angiogenesis during bone healing and repair [35, 36]. The HUVEC migration capacity in the CG, CG/PHA and CG/PHA/5%PBT groups was higher than that in the control group (*P* < 0.05), because the co-culture medium from macrophages and the CG, CG/PHA and CG/PHA/5%PBT hydrogels had a positive effect on cell migration. This is likely because these hydrogels can promote the secretion of BMP-2, VEGF, and bFGF in macrophages (Additional file 2: Fig. S4). The co-culture medium from macrophages and the CG/PHA/5%PBT piezoelectric hydrogels had the best effect with regard to promoting HUVEC migration and infiltration. Piezoelectric hydrogels display substantial potential for bone angiogenesis in bone regeneration. These data indicate that the CG/PHA/5%PBT piezoelectric hydrogels may provide an excellent electrical microenvironment for bone vascularization.

The ability to form blood vessels after bone injury plays a critical role in the reconstruction of bone defect tissue as they provide blood, nutrients, and oxygen to the damaged tissue and alleviate uncontrolled inflammatory responses to the damaged site [34, 37, 38]. Hence, a Matrigel tube formation assay was performed to assess the ability of the CG/PHA/PBT piezoelectric hydrogels to promote new vessel formation. HUVECs co-cultured with the co-culture medium from macrophages and the piezoelectric hydrogels showed more vascular tubes (Fig. 4d). However, a few incomplete tube-like structures were seen in the CG and CG/PHA groups. The percentage of blood vessel area and the total number of junctions were significantly increased in the CG/PHA/5%PBT group (*P* < 0.05, Fig. 4d), suggesting that CG/PHA/5%PBT piezoelectric hydrogels combined with macrophages could promote angiogenesis in vitro. All these results indicate that the co-culture medium from macrophages and the CG/PHA/5%PBT piezoelectric hydrogels could promote HUVEC migration and blood vessel formation.

The *VEGF*, *HIF-1α*, *bFGF*, and *Ang-1* genes are angiogenesis-related genes whose expression profile is closely related to angiogenesis [31, 39]. When the HUVECs were co-cultured with co-culture medium from macrophages and the piezoelectric hydrogels, the CG/PHA/5%PBT group showed significantly up-regulated expression of these genes compared with the control, CG and CG/PHA groups (*P* < 0.05, Fig. 4e), suggesting that the CG/PHA/PBT piezoelectric hydrogel had good angiogenic bioactivity in vitro. The highest expression of the angiogenesis-related genes was observed in the CG/PHA/5%PBT group, followed by the CG/PHA, CG, and control groups. There were no significant difference between the control and CG groups (*P* > 0.05). CD31 and VEGF double-immunofluorescence staining was further performed after 1 week of co-culture to assess the angiogenic effect of the CG/PHA/5%PBT piezoelectric hydrogel. As demonstrated in Fig. 4f, CD31 and VEGF expression in HUVECs on the CG/PHA/5%PBT group was significantly higher than that in the control, CG and CG/PHA groups. The immunofluorescence staining results were consistent with the RT-qPCR analysis. Therefore, the regulation of the immune microenvironment by piezoelectric hydrogel can effectively promote angiogenesis.

### Effects of the piezoelectric hydrogels on osteogenesis

To evaluate the effect of the co-culture medium from macrophages and the piezoelectric hydrogels on osteoblasts, many biological function assessments, such as cell migration, osteo-differentiation, and extracellular matrix (ECM) mineralization, were performed (Additional file 2: Fig. S6a). Both Transwell and scratch wound healing assays were used to analyze the migration of osteoblast (MC3T3-E1) cells treated with co-culture medium from macrophages and the piezoelectric hydrogels. As depicted in Additional file 2: Fig. S6b-c, the migration of MC3T3-E1 cells was highest in the CG/PHA/5%PBT group, while the migration in the CG and CG/PHA groups was slightly higher than that in the control group (*P* < 0.05). These data revealed that the CG/PHA/5%PBT piezoelectric hydrogels could stimulate MC3T3-E1 cell migration.

The osteogenic differentiation of MC3T3-E1 cells in the co-culture medium from macrophages and the piezoelectric hydrogels was evaluated by ALP staining, ARS staining, RT-qPCR assay, and immunofluorescence staining. ALP is a bioindicator of osteogenic differentiation in the beginning stages and was used to evaluate differentiation by staining and quantitative evaluation. The photographs shown in Additional file 2: Fig. S6d indicate that the co-culture medium from macrophages and the CG/PHA/5%PBT piezoelectric hydrogels displayed the most intensified ALP staining and ALP activity on both days 7 and 14 when compared with the other three groups (*P* < 0.05). ARS staining was applied to examine the mineralization status of different treatment groups. As demonstrated in Additional file 2: Fig. S6e, all cells adhered to the osteoinductive medium and co-culture medium from macrophages and the piezoelectric hydrogels, showing positive staining results on day 14 and day 21. The CG/PHA/5%PBT group expressed the best osteogenic ability. OD values were measured to quantitatively evaluate the ARS staining using a microplate reader at 562 nm; the results were consistent with those from light microscopy.

In addition, the expression of osteogenic genes, such as *Runx2*, *Col-1*, *OPN*, and *OCN*, was evaluated to validate the pro-osteogenic differentiation of MC3T3-E1 cells in the co-culture medium from macrophages and the piezoelectric hydrogels. As demonstrated in Additional file 2: Fig. S6f, highly up-regulated expression of these genes was detected in the CG/PHA/5%PBT group relative to that in the control (24-well plates), CG, and CG/PHA groups on day 14 (*P* < 0.05). Immunofluorescence staining also confirmed that the MC3T3-E1 cells cultured in the co-culture medium from macrophages and the piezoelectric hydrogels expressed higher levels of Runx2 and OPN proteins than other groups (Additional file 2: Fig. S6g). All these findings demonstrate that the CG/PHA/5%PBT piezoelectric hydrogels possessed a satisfactory ability to promote the osteogenic differentiation of MC3T3-E1 cells.

### In vivo evaluation of bone regeneration capability

Regarding biological performance, the piezoelectric hydrogels profoundly enhanced osteogenesis and effectively induced the switching of pro-inflammatory M1 macrophages to the anti-inflammatory M2 phenotype through multiple biochemical and biophysical properties. To further evaluate the applicability of the piezoelectric hydrogels for bone repair, we prepared a rat skull defect model according to a previously described method [18]. The hydrogels (CG, CG/PHA, and CG/PHA/5%PBT piezoelectric hydrogels) were implanted in the defect sites. The rats that did not receive any hydrogel comprised the blank control group. The piezoelectric hydrogels implanted in the bone injury sites could build a barrier in these sites. Subsequent neovascularization in the defect site eventually led to large-sized bone regeneration.

Macrophage polarization in the defect sites was detected by immunohistochemical staining 2 weeks after implantation. As shown in Additional file 2: Fig. S7a, the in situ bone tissue defect revealed a lower proportion of iNOS-positive cells and a higher proportion of CD206-positive cells in the CG/PHA/5%PBT group, indicating the development of an anti-inflammatory response following the implantation of the piezoelectric hydrogels. TNF-α, a common pro-inflammatory factor, and IL-10, an anti-inflammatory factor, are tightly linked to the inflammatory reaction at the injured site. Hence, immunohistochemical assays for TNF-α and IL-10 were performed to investigate the initial inflammatory reaction in the bone repair process. In comparison to the CG, CG/PHA, and CG/PHA/5%PBT groups, the pro-inflammatory factor TNF-α was significantly higher in the control group. In contrast, the anti-inflammatory factor IL-10 was up-regulated in the CG/PHA/5%PBT group (Additional file 2: Fig. S7b). These data confirmed that the CG/PHA/5%PBT hydrogel was more conducive to the M1-to-M2 shift of macrophages than the CG and CG/PHA hydrogel scaffolds.

By regulating the M1-to-M2 polarization of macrophages, the inflammatory response surrounding bone regeneration in situ can be effectively reshaped. Bone tissue regeneration following immune regulation should also be initiated. VEGF and BMP-2 function as potent inducers of angiogenesis and osteogenesis and are always secreted by M2 macrophages in the bone vascularization stage [40]. To verify the role of the CG/PHA/PBT piezoelectric hydrogel in the induction of angiogenesis and osteogenesis, immunohistochemical analysis of VEGF and BMP-2 was performed 2 weeks after surgery. Higher expression of VEGF and BMP-2 was observed in the CG/PHA/5%PBT group (Additional file 2: Fig. S7b), demonstrating greater potential for facilitating vascularization and bone formation. Moreover, immunofluorescence staining of CD31 and Runx2 was also performed 2 weeks after surgery to explore the angiogenic and osteogenic capabilities of the piezoelectric hydrogels. Higher expression of CD31 and Runx2 was observed in the CG/PHA/5%PBT group (Additional file 2: Fig. S7c), demonstrating greater potential for facilitating vascularization and bone remodeling. The electrical cues produced by the piezoelectric hydrogels could be effective for the transformation of the macrophage M1 phenotype to the M2 phenotype by enhancing VEGF, CD31, BMP-2, and Runx2 expression, which could create an accommodative microenvironment for osteogenic differentiation and immunomodulation. Therefore, the bone immunoregulatory microenvironment induced by piezoelectric hydrogels can differentiate endogenous mesenchymal stem cells (MSCs) into osteoblasts and enable them to achieve fast bone regeneration. In conclusion, the piezoelectric hydrogel exhibited a considerable ability to facilitate cranial bone repair.

**Fig. 5:**
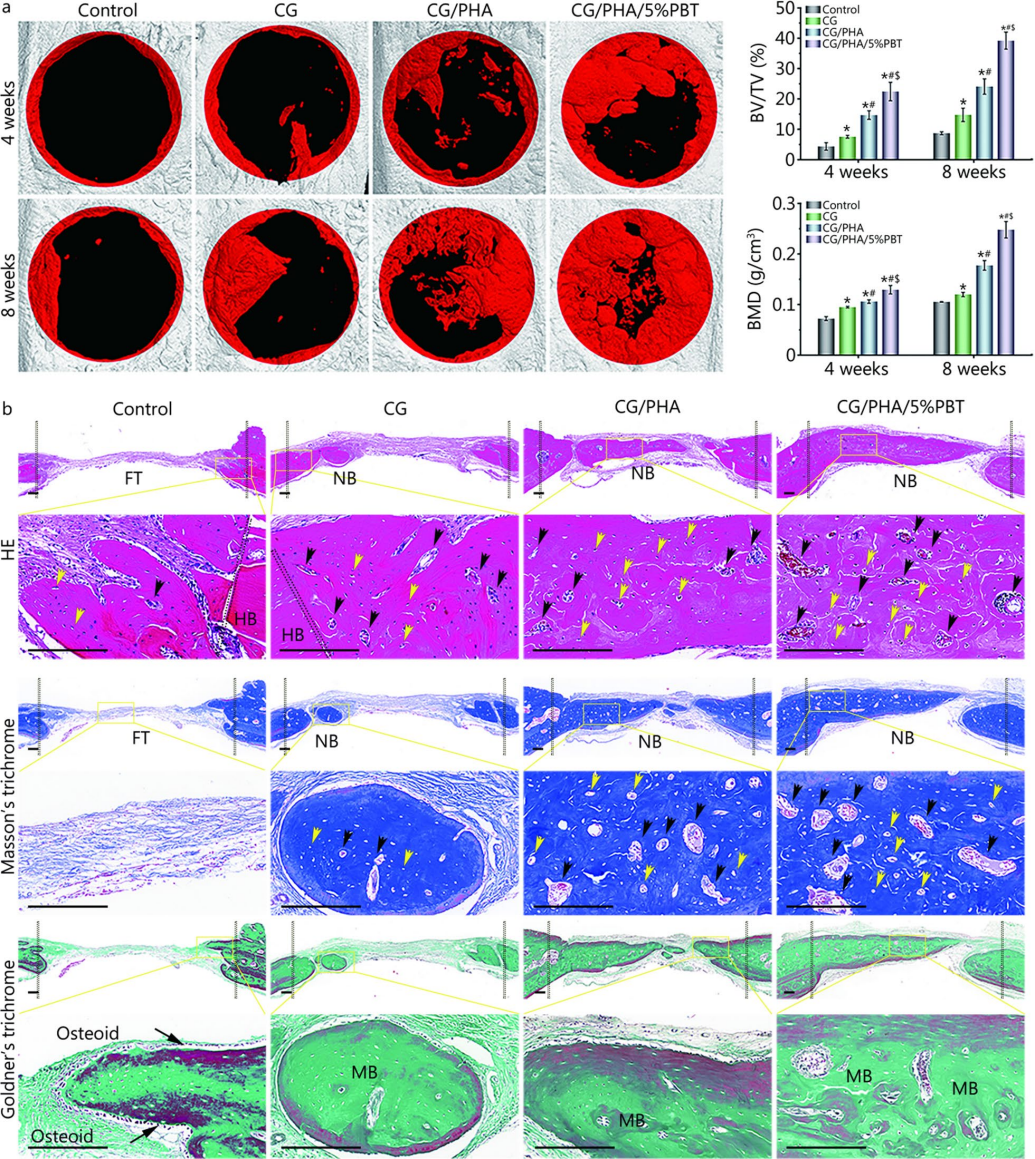
The CG/PHA/5%PBT piezoelectric hydrogel promotes bone tissue regeneration in vivo. **a** 2D micro-CT coronal and sagittal images, and the BV/TV and BMD results of regenerated bone tissues. Scale bar: 200 μm. **b** HE, Masson’s trichrome, and Goldner’s trichrome staining of the bone defect 8 weeks after implantation. Scale bar: 200 μm. Yellow arrows and black arrows represent the newly formed central canal and bone lacunae within the defect region, respectively. For Goldner’s trichrome staining images, a small island of immature bone (osteoid, red) was detected at the periphery of the defect site in the control group. Black dotted lines define the boundary of the critical-sized cranial bone defect. ^*^P < 0.05, compared with the control group; ^#^*P* < 0.05, compared with the CG group; ^$^*P* < 0.05, compared with the CG/PHA group. CG chitosan/gelatin, PHA polydopamine coated-hydroxyapatite, PBT polydopamine coated-barium titanate, BV/TV bone tissue volume/total tissue volume, BMD bone mineral density, FT fibrous tissue, NB newly formed bone tissue, HB host bone, MB mineralized/mature bone

To systematically test the osteogenesis induced by the piezoelectric hydrogel in a long profile, the newly formed bone was scanned by micro-CT. In Fig. 5a, the injured cranial bones to which the piezoelectric hydrogels were applied showed better repair than the control group both 4 and 8 weeks after implantation (*P* < 0.05). The data showed that much more new bone was formed after CG/PHA/5%PBT piezoelectric hydrogel implantation compared to the control, CG, and CG/PHA groups (*P* < 0.05). Eight weeks after implantation, the BV/TV of the CG/PHA/5%PBT group [(35.4 ± 4.1)%] was significantly higher than that of the CG/PHA group [(24.3 ± 5.2)%] and CG group [(15.8 ± 3.7)%] (*P* < 0.05). The sites implanted with the CG/PHA/5%PBT piezoelectric hydrogel also showed more mature bone with a higher density (*P* < 0.05). The BMD data showed the same tendencies as the BV/TV results. Collectively, the improved osteogenic behavior of the CG/PHA/5%PBT piezoelectric hydrogels was shown in cell experiments, and its enhancement in the pro-healing bone remodeling capability was also verified in a rat model.

Histological stainings, including HE staining, Masson’s and Goldner’s trichrome staining, were then performed to assess whether the piezoelectric hydrogel could enhance bone formation in vivo at 8 weeks (Fig. 5b). When the piezoelectric hydrogel was embedded for 8 weeks, the cambium tissue in the control group was mainly coarse fibrous tissue, with a small amount of scattered and discontinuous new bone. In contrast, the histological images demonstrated much denser bony tissues in the CG/PHA/5%PBT group than in the control, CG, and CG/PHA groups 8 weeks post-implantation. Unsurprisingly, the CG/PHA/5%PBT group had the highest amount of bone matrix formation among all the experimental groups and showed excellent bone regeneration 8 weeks after transplantation. Not only did the CG/PHA/5%PBT group regenerate the new bone lacunae and lamellar bone surrounded by the central canal, but the lamellar bone also expanded into the bone defect center to form a more complete bone structure. This result provides histological evidence that the continuous piezoelectric stimulation provided by the CG/PHA/5%PBT hydrogel after implantation recruited MSCs to the injured site and promoted their differentiation to osteoblasts. Notably, the top surface of the CG/PHA/5%PBT group was covered with a thin layer of reparative tissue 8 weeks post-implantation, suggesting the favorable compatibility and long-term biodegradability of our fabricated piezoelectric hydrogel in vivo. The red staining in Masson’s trichrome staining represents the mineralized collagen and mature bone matrix, while the blue staining depicts the non-mineralized collagen and immature bone matrix. In this study, the CG/PHA/5%PBT group showed abundant red staining, indicating that the regenerated skull in the hydrogel group was calcifying and that much of it was matured. In Goldner’s trichrome staining, the dark-green staining represents the mature lamellar bone and the red staining depicts the immature woven bone (osteoid). The Goldner’s trichrome staining results were consistent with the Masson’s trichrome staining results. Unexpectedly, the newly formed bone in the CG/PHA/5%PBT group was similar to the original bone. All in all, the above histochemical staining results indicate that the piezoelectric hydrogels have preeminent osteo-promotive capability.

Subsequently, immunohistochemical staining of Col-1, Runx2, OPN, CD31, and CD34 (osteogenesis-related and angiogenesis-related biomarkers) was performed to evaluate osteogenesis and angiogenesis in vivo. As shown in Additional file 2: Fig. S8, it was difficult to observe positive protein staining in the defect region of the control and CG groups at 8 weeks, while many patches of brown and/or reddish-brown staining were observed in the CG/PHA/5%PBT group, which indicates that the CG/PHA/5%PBT piezoelectric hydrogel could accelerate the expression of osteogenesis-related and angiogenesis-related genes. The expression of these proteins can continuously activate osteogenesis, angiogenesis, and ECM deposition. The high expression of Col-1, Runx2, OPN, CD31, and CD34 indicated that the bone defects covered with the CG/PHA/5%PBT piezoelectric hydrogel retained outstanding osteogenic and angiogenic capability.

### Possible molecular mechanism by which the hydrogels promote bone regeneration

**Fig. 6:**
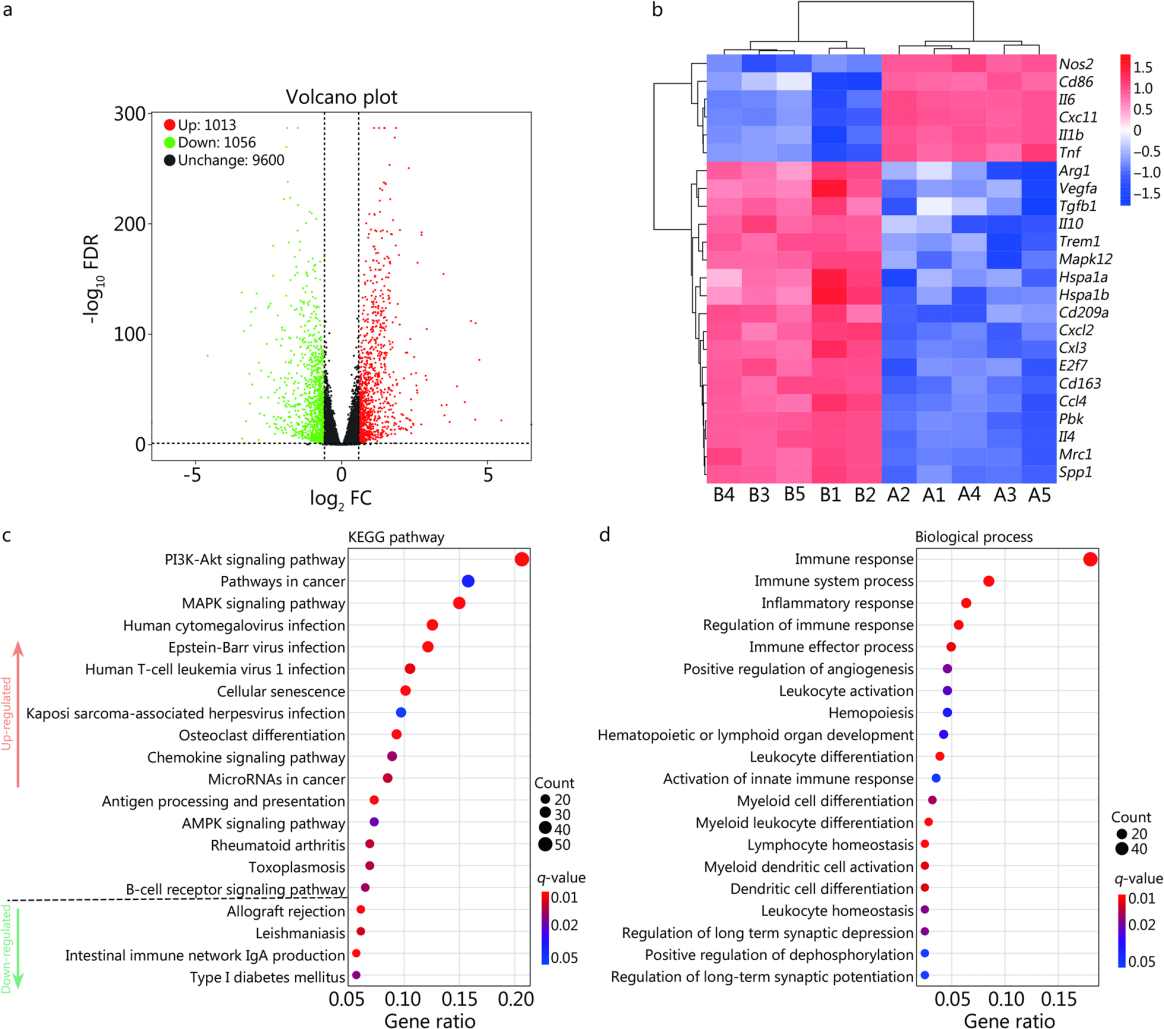
Transcriptome analysis of macrophage immunity regulated by the CG/PHA/5%PBT piezoelectric hydrogel. **a** Quantitative analysis of DEGs in macrophages from the CG and CG/PHA/5%PBT groups. **b** Heatmap of DEGs associated with immune regulation. A1–A5 are CG hydrogel samples, and B1–B5 are CG/PHA/5%PBT piezoelectric hydrogel samples. **c** KEGG pathway analysis in macrophages from the CG and CG/PHA/5%PBT groups. **d** GO enrichment analysis of the 20 most differentially up-regulated and down-regulated biological processes. CG chitosan/gelatin, PHA polydopamine coated-hydroxyapatite, PBT polydopamine coated-barium titanate, DEGs differentially expressed genes, KEGG kyoto encyclopedia of genes and genomes, GO gene ontology

Following the in vitro and in vivo analyses, the molecular mechanism underlying the effect of CG/PHA/5%PBT piezoelectric hydrogel treatment on macrophages was determined via RNA sequencing. A total of 2069 differentially expressed genes (DEGs), including 1013 up-regulated and 1056 down-regulated genes, were obtained between the CG/PHA/5%PBT group and the CG group (Fig. 6a). The heatmap of DEGs associated with immune regulation demonstrated that anti-inflammatory and pro-healing genes, such as *Arg1*, *Vegfa*, *Tgfb1*, *Il10*, *Trem1*, *Mapk12*, *Hspa1a*, *Hspa1b*, *Cd209a*, *Cxcl2*, *Ccl3*, *E2f7*, *Cd163*, *Ccl4*, *Pbk*, *Il4*, *Mrc1*, and *Spp1*, were overexpressed, while osteoclast differentiation was down-regulated in CG/PHA/5%PBT group (*P* < 0.05, Fig. 6b). Pro-inflammatory genes, such as *Nos2*, *Il6*, *IL1b*, *Tnf*, *Cxc11*, and *CD86*, were down-regulated in the CG/PHA/5%PBT group (*P* < 0.05, Fig. 6b). The KEGG database (http://www.genome.jp/kegg/) is a commonly used bioinformatics-based website for gene function analysis. In this study, a total of 11,729 unigenes were grouped into 258 known pathways in the KEGG database. The KEGG pathway results demonstrated that immune-related signaling pathways were overexpressed, such as the PI3K-Akt signaling pathway, MAPK signaling pathway, chemokine signaling pathway, antigen processing and presentation, AMPK signaling pathway, and B-cell receptor signaling pathway (Fig. 6c). Gene Ontology (GO) is another system for gene categorization. According to the detection results, 11,729 unigenes were classified and divided into three categories: molecular function, cellular component, and biological process. These unigenes were further divided into 58 subcategories (Additional file 2: Fig. S9a). The biological process enrichment analysis showed that the immune response-related genes were closely related (Fig. 6d). In the cellular component category, the Golgi apparatus was the most highly expressed unigene (Additional file 2: Fig. S9b). Within the molecular function category, the five most abundant subcategories were ATP binding, identical protein binding, binding, macromolecular complex binding, and protein kinase binding (Additional file 2: Fig. S9c). Based on the GO analysis, we presume that the CG/PHA/5%PBT piezoelectric hydrogel could regulate the immune response based on macrophages. The main function of the Golgi apparatus is to process, sort, and transport proteins synthesized by the endoplasmic reticulum and then send them to specific parts of the cell or secrete them outside the cell. Hence, the polarization of macrophages is closely related to the metabolism of the Golgi apparatus. At the molecular level, the process of macrophage polarization involves energy metabolism and dynamic membrane binding.

**Fig. 7:**
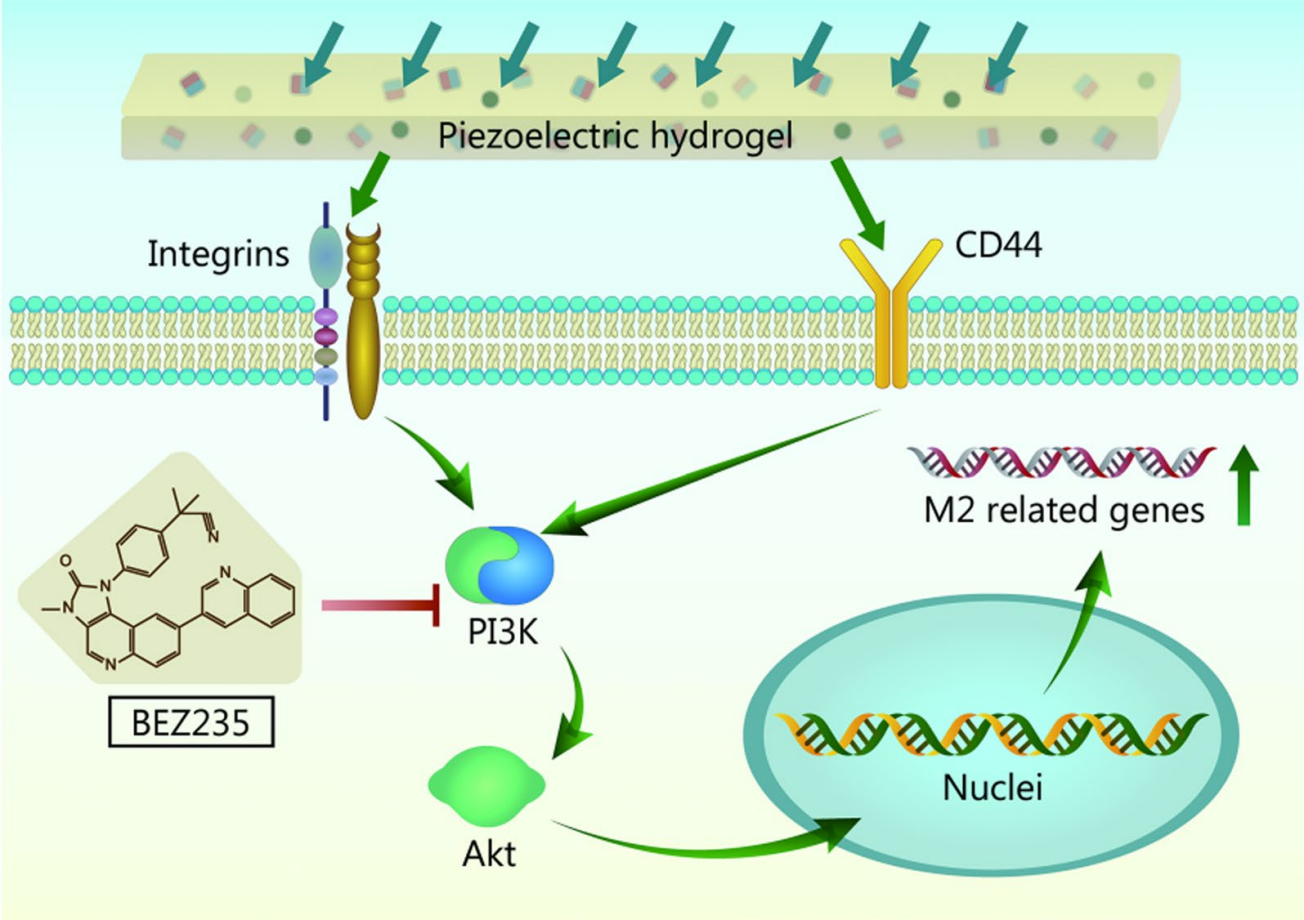
Schematic diagram of the possible molecular mechanism by which the CG/PHA/5%PBT piezoelectric hydrogel promotes bone repair by regulating macrophage M2 polarization and activating the PI3K/Akt axis. CG chitosan/gelatin, PHA polydopamine coated-hydroxyapatite, PBT polydopamine coated-barium titanate, PI3K phosphoinositol 3 kinase, Akt serine/threonine protein kinase

According to the RNA sequencing results, the PI3K-Akt signaling pathway may play a vital role in regulating macrophage M2 polarization. Since phosphorylated proteins are in an active state that regulates cell functions, we used immunohistochemistry to detect the levels of p-PI3K and p-Akt in regenerated bone tissues (Additional file 2: Fig. S10a). The results showed that the p-PI3K and p-Akt protein expression was highest in the CG/PHA/5%PBT group, followed by the CG/PHA and CG groups (*P* < 0.05). It can be inferred that the CG/PHA/5%PBT piezoelectric hydrogel could significantly activate the PI3K/Akt signaling axis to regulate macrophage M2 polarization. Based on the animal experiment results, we used the PI3K inhibitor BEZ235 (0, 10, 100, and 200 ng/ml) to block the PI3K/Akt signaling pathway in macrophages inoculated in the piezoelectric hydrogels. As shown in Additional file 2: Fig. S10b, macrophages could be switched to the M1 phenotype by adding LPS (200 ng/ml) to the co-culture system of piezoelectric hydrogels and macrophages. IL-4, an anti-inflammatory cytokine, could significantly promote macrophage type 2 polarization at a concentration of 20 ng/ml. In contrast, BEZ235, an inhibitor of PI3K, could significantly inhibit the M2 polarization of macrophages. When the concentration of the inhibitor BEZ235 decreased to 0 ng/ml, the inhibitory effect disappeared and the hydrogels could promote the M2 polarization of the macrophages. It has been reported that integrins and CD44 are upstream elements of the PI3K/Akt signaling pathway involved in the polarization of macrophages [41]. Hence, a hypothetical scheme of the mechanism by which the CG/PHA/5%PBT piezoelectric hydrogel regulates macrophage M2 polarization is proposed in Fig. 7.

## Discussion

In the present study, a versatile CG/PHA/PBT piezoelectric hydrogel scaffold was successfully constructed for bone tissue engineering. This piezoelectric hydrogel not only generates electricity spontaneously but also regulates immunity, angiogenesis, and osteogenesis. We have carefully reasoned out the mechanism underlying the spontaneous electricity of this piezoelectric hydrogel in Fig. 1c, which can explain how it creates a piezoelectric microenvironment conducive to regeneration in vivo. The pro-bone-formation effect of the piezoelectric hydrogels was verified by several in vitro and in vivo experiments. Moreover, we identified the key signaling pathway (PI3K/Akt axis) at the molecular level by transcriptome sequencing. Finally, we returned to in vivo experiments to verify the expression changes of phosphorylated PI3K and Akt proteins as well as in vitro inhibition experiments to determine their regulatory roles. Collectively, our results show that the self-powered CG/PHA/PBT piezoelectric hydrogel could regulate macrophage M2 polarization by creating a piezoelectric microenvironment, thus promoting angiogenesis and osteogenesis, and ultimately, bone healing.

Gel is a water-soluble protein that is produced by processing and denaturing collagen, which is the main protein of skin, tendons, bones, and other tissues [42]. It is precisely because of its excellent solubility that collagen has poor mechanical properties, which makes its use in tissue engineering alone difficult. As a result, collagen usually needs to be combined with other polymers and chemically cross-linked before it can be used in tissue engineering [43]. Cs is a kind of natural cationic polysaccharide that is generally obtained by deacetylation of the exoskeleton of crustaceans and insects [44]. When Cs was chemically cross-linked with Gel, the mechanical properties of the CG hydrogel improved to a large extent [45, 46]. Prokhorov et al. [47] fabricated a CS-BT piezoelectric polymer, the in vitro experiments verified the possibility in tissue engineering. Zhang et al. [48] developed CG-based sutures. In vivo experiments showed that these CG-based sutures can reduce the inflammatory response and promote tissue integration due to the synergistic effect of porous structure and surface bioactive coating, which has great potential for application in tendon healing and functional remodeling. Park et al. [49] have proposed an unconventional ribbon filter material viscoelastic CG hydrogel damper to selectively remove dynamic mechanical noise artifacts. Hydrogels exhibit frequency-dependent phase transitions resulting in a rubber state that dampens low-frequency noise and a glass state that transmits the desired high-frequency signal. The CG hydrogels demonstrated an adaptive pass filter that captures high-quality signals from patients while minimizing signal processing in advanced bioelectronics.

At present, most experiments on improving bone regeneration mainly focus on the following two aspects: 1) promoting the differentiation of osteoblasts and 2) reducing the number and activity of osteoclasts. The current mainstream research direction is to enhance the differentiation of osteoblasts, for example, by increasing the growth factors directly targeting osteoblasts, such as BMP-2 and Runx2 [6, 50–53]. HA can realize the combination of chemical bonds with the bone tissue interface and has a certain solubility in the human body. The release of ions can participate in bone metabolism in the body and has a stimulatory and inducing effect on bone hyperplasia or osteogenic differentiation. A large number of cellular and animal experiments have shown that HA has superior bone differentiation activity [54, 55].

BaTiO_3_ nanoparticles have shown great promise in the biomedical engineering field [9, 56, 57]. Kim et al. [58] demonstrated that BaTiO_3_ can be used to stimulate brain deep tissue by producing nitric oxide (NO) and generating electric current under ultrasound. The release of NO could break the blood–brain barrier temporarily and allow BaTiO_3_ nanoparticles to accumulate in the brain parenchyma, and the piezo-induced output current stimulates dopaminergic neuron-like cells to release dopamine. The application of BaTiO_3_ can cause remission of the symptoms of Parkinson’s disease without significant toxicity. Liu et al. [59] proposed a BaTiO_3_ piezocatalytic hydrogel to promote bacterial-infected wound healing. The BaTiO_3_ composite hydrogel has a strong internal electric field and produces ROS due to the presence of BaTiO_3_ nanoparticles, which have excellent antibacterial effects that help heal infected wounds. Therefore, BaTiO_3_ nanoparticles show great prospects for application in the field of biomedical engineering due to their excellent piezoelectric properties. Due to the comprehensive assessment of the properties of Cs, Gel, HA, and BaTiO_3_, we use this quaternary complex for bone tissue regeneration research.

The BaTiO_3_ hydrogel in this study worked as nanogenerators and could generate a 0.8 V voltage output under 1 kPa pressure. Sun et al. [60] developed a wood sponge piezoelectric nanogenerator that generated a 0.4 V voltage output under 4.4 kPa pressure. In addition, a series of Cs nanogenerators just generated a 0.06 V voltage output [61]. Hence, our BaTiO_3_ hydrogel nanogenerator exhibits more excellent self-powered performance. In military medicine, the piezoelectric hydrogel could not only be used as a scaffold for bone tissue engineering, but also would work as a biosensor and energy storage device [62, 63], due to the self-powered property of the piezoelectric hydrogel.

Repair after bone injury involves multiple biological reaction stages: 1) In the early stage of the inflammatory response, trauma-related signals can activate immune cells, leading to changes in the chemical gradients of inflammatory factors and the recruitment of more immune cells to the site of bone injury, thus triggering the immune response; 2) Chronic inflammation: 3–5 d after injury, necrotic tissues and bacteria can be removed through acute inflammation, and the bone defect site then enters a chronic inflammation stage. In this stage, macrophages at the defect site gradually become polarized toward the M2 phenotype (anti-inflammatory type), and the body induces stem cells to differentiate into various functional cells through immune regulation and recruitment, promoting the production of new bone tissues [64, 65]. The inflammatory microenvironment can be transformed into a repair microenvironment by the secretion of regenerative cytokines such as Runx2 and TGF-β [66]. Therefore, how to promote the polarization of macrophages to the M2 type in bone repair is an urgent problem to be addressed in bone regeneration. Many reports have shown that the endogenous electrical microenvironment of primary bone tissue can be reproduced by preparing composite piezoelectric materials [9, 15, 67]. When the CG/PHA/PBT piezoelectric hydrogel was applied to the macrophages, the macrophages transformed to the M2 phenotype.

In addition, we screened the key signaling pathways by which the piezoelectric hydrogel regulates macrophage polarization through transcriptional tests. The inhibition tests could verify that CG/PHA/PBT hydrogels up-regulate PI3K/Akt phosphorylation to regulate macrophage M2 polarization. In the schematic diagram of Fig. 7, we summarized the mechanism of piezoelectric hydrogel through in vitro and in vivo experiments. The piezoelectric microenvironment and stimulation caused by the piezoelectric hydrogel act on the macrophage surface receptors, then it leads to the activation of the downstream signaling pathway to regulate the positive regeneration microenvironment. Liu et al. [68] stated that integrin β1 is an upstream signaling molecule of PI3K/Akt, and their developed biological scaffold materials can regulate the polarization of macrophages through the activation of integrin β1 macrophage surface receptors to activate the PI3K/Akt signaling pathway. Duan et al. [41] found that CD44 and Rho-associated protein kinase (ROCK) are upstream molecules of PI3K/Akt, while mammalian target of rapamycin (mTOR) is the downstream protein of PI3K/Akt that regulates the polarization of macrophages. Combined with our results, these findings demonstrate the importance of the PI3K/Akt signaling pathway in macrophage polarization. In addition to this, Ma et al. [69] stated that the Toll‑like receptor (TLR) signaling pathway is involved in the regulation of macrophage polarization via the modular analysis and functional enrichment analysis. Besides, activating Notch signaling can regulate the differentiation of macrophages into M1 and play a role in promoting inflammation and antitumor activity [70]. These results and some other reports provide key protein targets for us to develop scaffolds and drugs to promote bone tissue regeneration. In addition, we only applied this piezoelectric hydrogel to bone repair in this research, and we would expand the application of this kind of piezoelectric hydrogel to nerve, ligament, muscle and skin tissue engineering research in future studies.

## Conclusions

In this study, we designed and synthesized a versatile porous piezoelectric hydrogel that has good immunoregulatory, angiogenic, and osteogenic functions and can rapidly realize new bone formation. We introduced PHA and PBT nanoparticles into the hydrogel, which can not only improve the physical and chemical properties and biocompatibility of the hydrogel but also promote osteogenic activity. This hydrogel scaffold can significantly promote the adhesion, proliferation, and osteogenesis of osteoblast (MC3T3-E1). Since the piezoelectric hydrogel can continuously generate endogenous electrical stimulation, it has corresponding immunomodulatory effects, which can synergistically promote bone regeneration and vascular network reconstruction. Several experiments verified that the versatile piezoelectric hydrogel created a pro-healing immune niche. Many anti-inflammatory macrophages (M2 phenotype) and few pro-inflammatory macrophages (M1 phenotype) were observed in the piezoelectric hydrogel-treated bone defect site. The CG/PHA/PBT piezoelectric hydrogel activates the PI3K/Akt signaling axis to regulate macrophage M2 polarization. Thus, we investigated in detail the ability of the hydrogel to promote M2 macrophage phenotype polarization, improve osteogenic differentiation and angiogenesis, and accelerate bone regeneration and remodeling in this study. The prepared piezoelectric hydrogel implant is one kind of potential bioactive scaffold biomaterial with a special ability to generate a piezoelectric microenvironment. According to these observations, the CG/PHA/PBT piezoelectric hydrogel bone tissue scaffold shows potential as a bone graft scheme for clinical application in large bone defect repair.

## Supplementary Information

**Additional file 1: Table S1** The primer sequences of each gene.

**Additional file 2: Fig. S1** Flow chart of animal experiments and corresponding tests. **Fig. S2** Transmission electron microscopy (TEM) images of barium titanate nanoparticles. **Fig. S3** Analysis of piezoelectric hydrogel promoting macrophage polarization. **Fig. S4** Pro-healing cytokine secretion in macrophages stimulated by piezoelectric hydrogels. **Fig. S5** Representative quantitative analysis of the migratory ability of HUVEC cells in the Transwell (**a**) and wound healing migration (**b**) assay for Fig. 4b-c. **Fig. S6** In vitro evaluation of osteogenic activity. **Fig. S7** In vivo evaluation of immune regulation; angiogenesis, and osteogenesis after piezoelectric hydrogel implantation. **Fig. S8** Representative immunohistochemical staining images of Col-1, Runx2, OPN, CD31, and CD34 within the defect area 8 weeks after implantation. **Fig. S9** Results of transcriptome sequencing of macrophage under the piezoelectric hydrogel stimulation. **Fig. S10** The CG/PHA/5%PBT piezoelectric hydrogel regulates bone repair via the PI3K/Akt axis.

## Data Availability

The data supporting this article can be found within the text and the supplementary information file. Any additional data and the data supporting the plots within this paper are available from the corresponding author upon reasonable request.

## Abbreviations

AC: Alternating current
ALP: Alkaline phosphatase
AR: Alizarin red
BT: Barium titanate
BMP-2: Bone morphogenetic protein 2
Cs: Chitosan
cDNA: Complementary DNA
Col-1: Collagen type I
EDTA: Ethylene diamine tetraacetic acid
Gel: Gelatin
HUVECs: Human umbilical vein endothelial cells
HA: Hydroxyapatite
HE: Hematoxylin and eosin
iNOS: Inducible nitric-oxide synthase
KEGG: Kyoto Encyclopedia of Genes and Genomes
MSCs: Mesenchymal stem cells
OPN: Osteopontin
OCN: Osteocalcin
PDA: Polydopamine
PHA: Polydopamine-modified ceramic hydroxyapatite
ROS: Reactive oxygen species
Runx2: Runt-related transcription factor 2
TLR: Toll‑like receptor
TEM: Transmission electron microscopy
VEGF: Vascular endothelial growth factor
