# Supplementary material for: The marriage of immunomodulatory, angiogenic, and osteogenic capabilities in a piezoelectric hydrogel tissue engineering scaffold for military medicine
Source: Mil Med Res. 2023 Jul 31;10:35. doi: 10.1186/s40779-023-00469-5 (PMC10388535; doi:10.1186/s40779-023-00469-5)
Supplement: Supplementary file 1 — Additional file 1: Table S1 The primer sequences of each gene. [file 40779_2023_469_MOESM1_ESM.pdf]

Table S1 The primer sequences of each gene

Genes	Primers	Application
IL-6	Forward: GAGACCACTGGGGAGAATGC<br>Reverse: TTGCCAGGTGGGTAAAGTGG	RAW 264.7
TNF-α	Forward: CAGGCGGTGCCTATGTCTC<br>Reverse: CGATCACCCCGAAGTTCAGTAG	
iNOS	Forward: GAATCTTGGAGCGAGTTG<br>Reverse: CCAGGAAGTAGGTGAGGG	
CD86	Forward: ATGGGCTCGTATGATTGT<br>Reverse: TCTTAGGTTTCGGGTGAC	
IL-4	Forward: CATCCTGCTCTTCTTTCTC<br>Reverse: TTCTCCTGTGACCTCGTT	
IL-10	Forward: TTTCAAACAAAGGACCAG<br>Reverse: GGATCATTTCCGATAAGG	
Arg-1	Forward: AAGACAGCAGAGGAGGTG<br>Reverse: AGTCAGTCCCTGGCTTAT	
CD206	Forward: GCAAGTGATTTGGAGGCT<br>Reverse: ATAGGAAACGGGAGAACC	
GAPDH	Forward: TCAACGGCACAGTCAAGG<br>Reverse: TTAGTGGGGTCTCGCTCC	
VEGF	Forward: TATGCGGATCAAACCTCACCA<br>Reverse: CACAGGGATTTTTCTTGTCTTGCT	HUVECs
HIF-1α	Forward: ATCCATGTGACCATGAGGAAAT<br>Reverse: CTCGGCTAGTTAGGGTACACTT	
bFGF	Forward: AAGAGCGACCCTCACATCAA<br>Reverse: GCCAGGTAACGGTTAGCACA	
Ang-1	Forward: CAGGAGGATGGTGGTTTG<br>Reverse: GCCCTTTGAAGTAGTGCC	
GAPDH	Forward: CATCATCCCTGCCTCTACTGG<br>Reverse: GTGGGTGTCGCTGTTGAAGTC	
Runx2	Forward: AATCCACAAGGACAGAGTCAGAT	

	Reverse: ACTGCCTGGGGTCTGAAAAAG	
Col-1	Forward: ACGCCATCAAGGTCTACTGC	
	Reverse: ACTCGAACGGGAATCCATCG	
OPN	Forward: CATTCTCGGAGGAAACCAGC	
	Reverse: GAATTCAGCCAGGAGAACTGC	MC3T3-E1
OCN	Forward: TTTCTGCTCACTCTGCTGACC	
	Reverse: ATGCGTTTGTAGGCGGTCTT	
GAPDH	Forward: TGAAGGGTGGAGCCAAAAG	
	Reverse: AGTCTTCTGGGTGGCAGTGAT	

IL-6 interleukin-6, *TNF-α* tumor necrosis factor-α, *iNOS* inducible nitric-oxide synthase, *IL-4* interleukin-4, *IL-10* interleukin-10, *Arg-1* arginase 1, *CD206* mannose receptor, *VEGF* vascular endothelial growth factor, *HIF-1α* hypoxia inducible factor-1α, *bFGF* basic fibroblast growth factor, *Ang-1* angiopoietin-1, *Runx2* Runt-related transcription factor 2, *Col-1* collagen type I, *OPN* osteopontin, *OCN* osteocalcin, *HUVECs* human umbilical vein endothelial cells
