# Supplementary material for: The marriage of immunomodulatory, angiogenic, and osteogenic capabilities in a piezoelectric hydrogel tissue engineering scaffold for military medicine
Source: Mil Med Res. 2023 Jul 31;10:35. doi: 10.1186/s40779-023-00469-5 (PMC10388535; doi:10.1186/s40779-023-00469-5)
Supplement: Supplementary file 2 — Additional file 2: Fig. S1 Flow chart of animal experiments and corresponding tests. Fig. S2 Transmission electron microscopy (TEM) images of barium titanate nanoparticles. Fig. S3 Analysis of piezoelectric hydrogel promoting macrophage polarization. Fig. S4 Pro-healing cytokine secretion in macrophages stimulated by piezoelectric hydrogels. Fig. S5 Representative quantitative analysis of the migratory ability of HUVEC cells in the Transwell (a) and wound healing migration (b) assay for Fig. 4b-c. Fig. S6 In vitro evaluation of osteogenic activity. Fig. S7 In vivo evaluation of immune regulation; angiogenesis, and osteogenesis after piezoelectric hydrogel implantation. Fig. S8 Representative immunohistochemical staining images of Col-1, Runx2, OPN, CD31, and CD34 within the defect area 8 weeks after implantation. Fig. S9 Results of transcriptome sequencing of macrophage under the piezoelectric hydrogel stimulation. Fig. S10 The CG/PHA/5%PBT piezoelectric hydrogel regulates bone repair via the PI3K/Akt axis. [file 40779_2023_469_MOESM2_ESM.pdf]

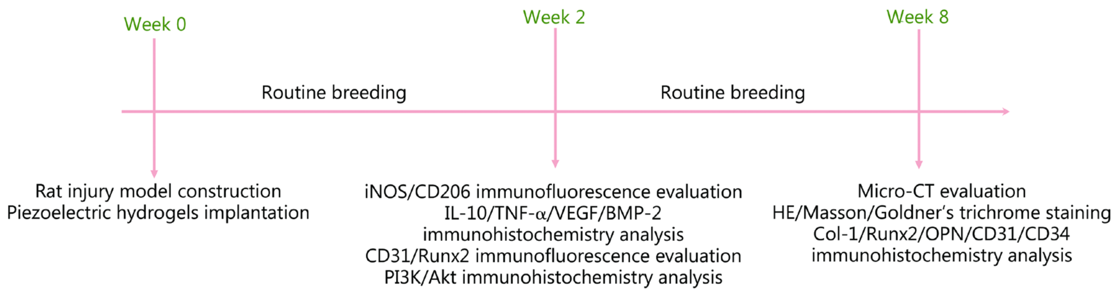

Fig. S1 Flow chart of animal experiments and corresponding tests.

iNOS inducible nitric-oxide synthase, CD206 mannose receptor, IL-10 interleukin-10, TNF-α tumor necrosis factor-α, VEGF vascular endothelial growth factor, BMP-2 bone morphogenetic protein 2, Col-1 collagen type I, Runx2 Runt-related transcription factor 2, OPN osteopontin, CD31 platelet endothelial cell adhesion molecule-1, CD34 hematopoietic progenitor cell antigen

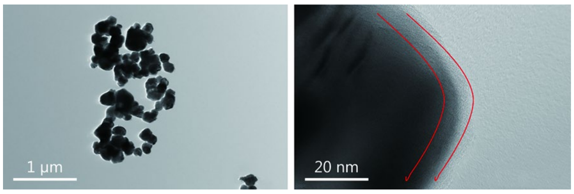

Fig. S2 Transmission electron microscopy (TEM) images of barium titanate nanoparticles.

The middle of the red line shows the dopamine coating

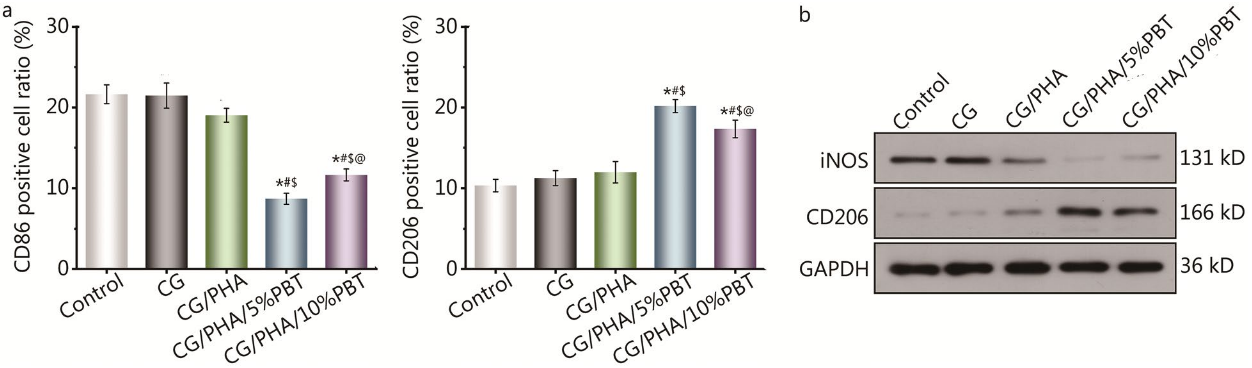

Fig. S3 Analysis of piezoelectric hydrogel promoting macrophage polarization.

a Statistical results of the flow cytometric dot plots from **Fig. 3f**. **b** Representative Western blotting images of the M1 macrophage marker iNOS and the M2 macrophage marker CD206 in RAW 264.7 cells cultured on various hydrogel samples on day 2. $^{*}P < 0.05$, compared with the control group; $^{\#}P < 0.05$, compared with the CG group; $^{\$}P < 0.05$, compared with the CG/PHA group; $^{@}P < 0.05$, compared with the CG/PHA/5%PBT group. $n = 3$. CG chitosan/gelatin, PHA polydopamine coated-hydroxyapatite, PBT polydopamine coated-barium titanate, iNOS inducible nitric-oxide synthase, CD206 mannose receptor

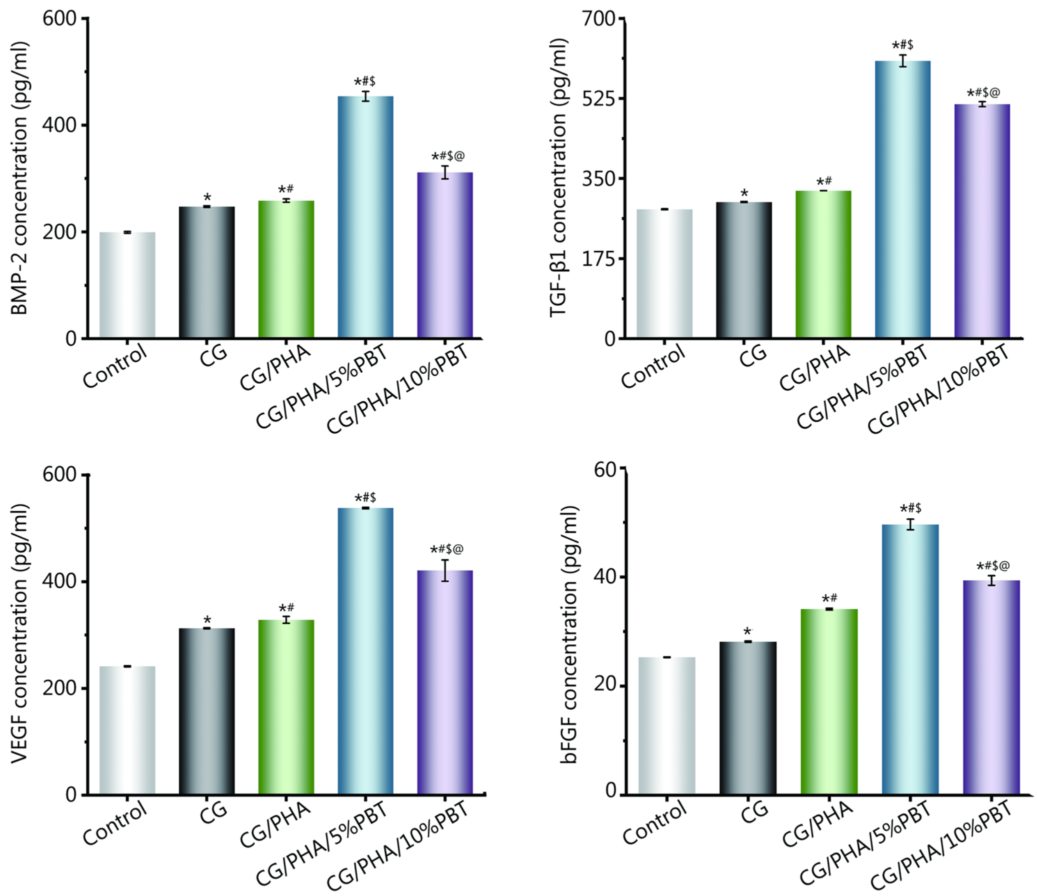

Fig. S4 Pro-healing cytokine secretion in macrophages stimulated by piezoelectric hydrogels. BMP-2, TGF-β1, VEGF and bFGF secreted from macrophages stimulated by piezoelectric hydrogels. $^{*}P < 0.05$, compared with the control group; $^{\#}P < 0.05$, compared with the CG group; $^{\$}P < 0.05$, compared with the CG/PHA group; $^{@}P < 0.05$, compared with the CG/PHA/5%PBT group. $n = 3$. BMP-2 bone morphogenetic protein 2, TGF-β1 transforming growth factor beta 1, VEGF vascular endothelial growth factor, bFGF basic fibroblast growth factor, CG chitosan/gelatin, PHA polydopamine coated-hydroxyapatite, PBT polydopamine coated-barium titanate

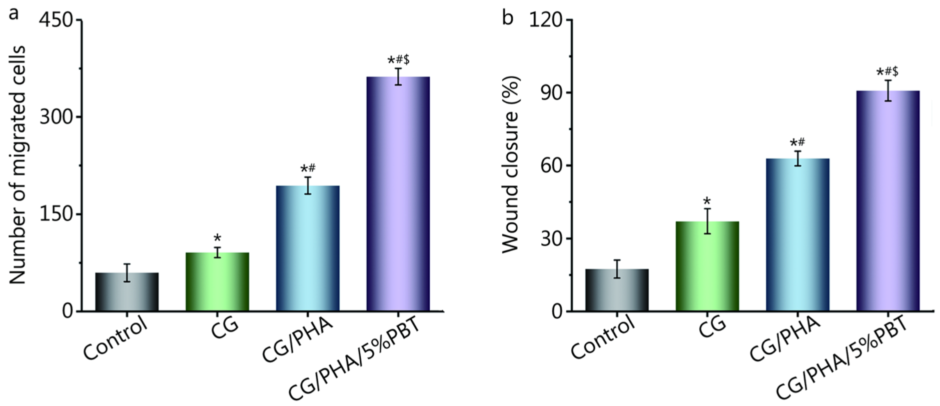

Fig. S5 Representative quantitative analysis of the migratory ability of HUVEC cells in the Transwell (**a**) and wound healing migration (**b**) assay for **Fig. 4b-c**.

$^{*}P < 0.05$, compared with the control group; $^{\#}P < 0.05$, compared with the CG group; $^{\$}P < 0.05$, compared with the CG/PHA group. $n = 3$. CG chitosan/gelatin, PHA polydopamine coated-hydroxyapatite, PBT polydopamine coated-barium titanate, HUVEC human umbilical vein endothelial cell

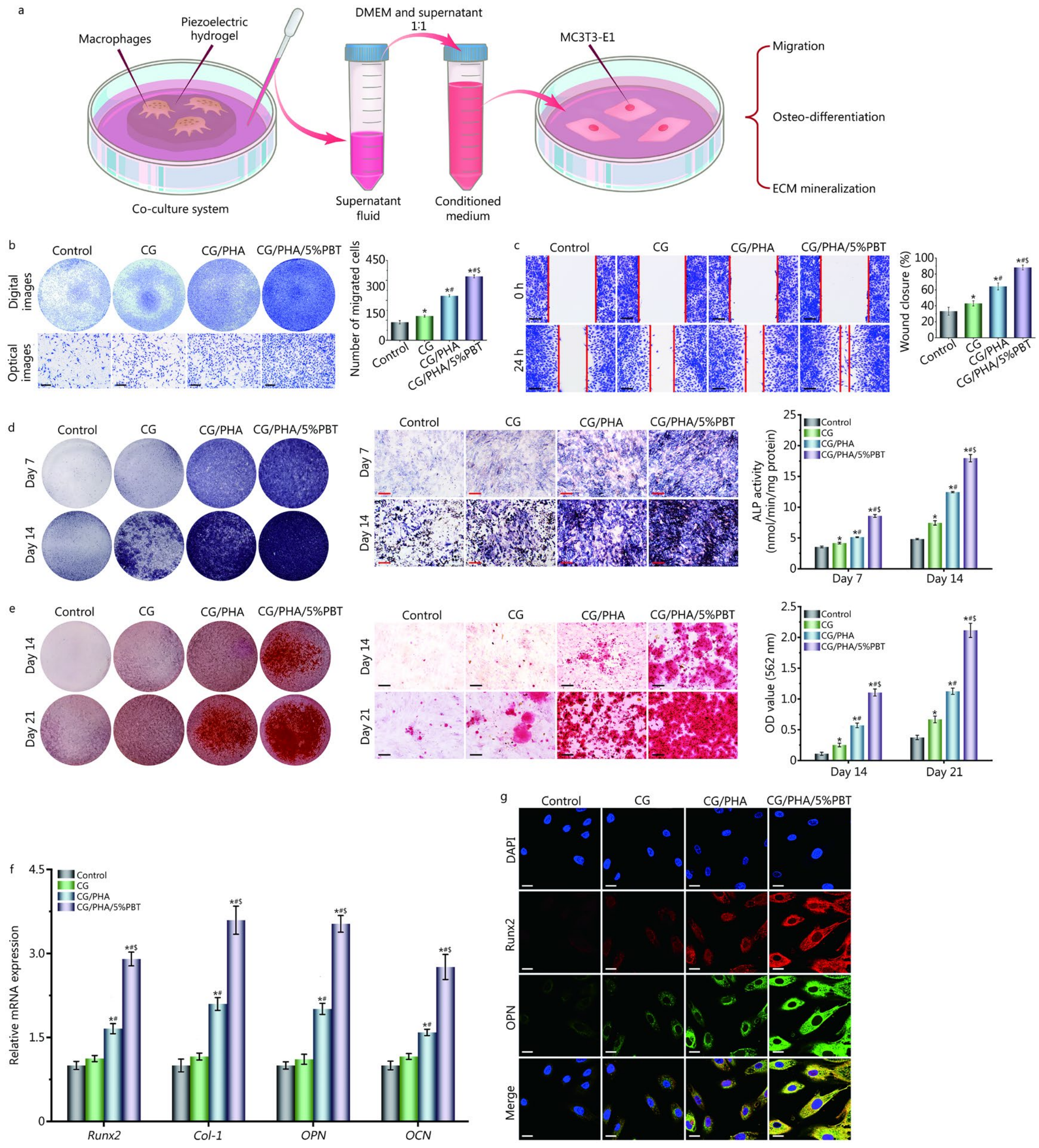

Fig. S6 In vitro evaluation of osteogenic activity.

a Schematic diagram of the cell experiment showing the promotion of osteogenic differentiation. **b** Representative Transwell migration images and quantitative analysis of the migratory ability of MC3T3-E1 cells in the Transwell assay. Scale bar: 200 μm. **c** Representative wound healing migration images and quantitative analysis of the migratory ability of MC3T3-E1 cells in the scratch assay. Scale bar: 200 μm. **d** Representative ALP staining assays for MC3T3-E1 cells incubated with different scaffold extracts for 7 days and 14 days, and the quantitative analysis of ALP activity staining in different groups. Scale bar: 200 μm. **e** Representative ARS staining assays for MC3T3-E1 cells incubated with different scaffold extracts for 14 days and 21 days, and quantitative analysis of ARS staining in different groups. Scale bar: 200 μm. **f** mRNA expression

of osteogenesis-related genes *Runx2*, *Col-1*, *OPN* and *OCN*. The expression of these osteogenesis-related genes increased significantly under hydrogel piezoelectric stimulation. **g** Immunofluorescence image of Runx2 (red) and OPN (green), the expression of these osteogenesis-related protein increased significantly under hydrogel piezoelectric stimulation. Scale bar: 25 μm. $^{*}P < 0.05$, compared with the control group; $^{\#}P < 0.05$, compared with the CG group; $^{\$}P < 0.05$, compared with the CG/PHA group; $^{@}P < 0.05$, compared with the CG/PHA/5%PBT group. $n = 3$. DMEM Dulbecco's modified Eagle's medium, CG chitosan/gelatin, PHA polydopamine coated-hydroxyapatite, PBT polydopamine coated-barium titanate, ECM extracellular matrix, ALP alkaline phosphatase, ARS alizarin red S, Runx2 Runt-related transcription factor 2, Col-1 collagen type I, OPN osteopontin, OCN osteocalcin

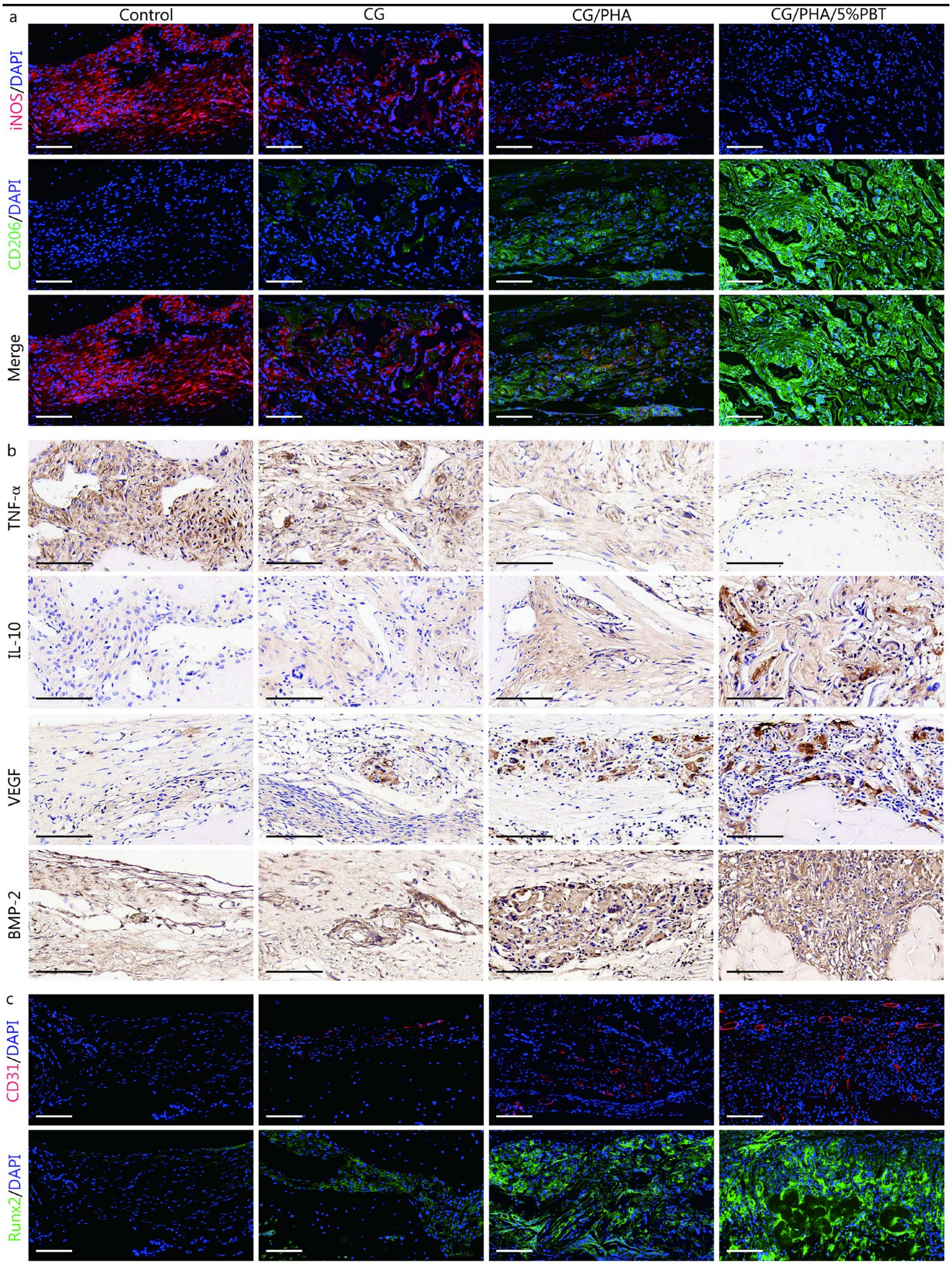

Fig. S7 In vivo evaluation of immune regulation, angiogenesis, and osteogenesis after piezoelectric hydrogel implantation.

a Representative immunofluorescence staining images of iNOS and CD206 within the defect area 2 weeks after implantation. Scale bar: 100 μm. **b** Representative immunohistochemical staining images of TNF-α, IL-10, VEGF, and BMP-2 within the defect area 2 weeks after implantation. Scale bar: 100 μm. **c** Representative CD31 and Runx2 staining of the bone defect 2 weeks after

implantation. Scale bar: 100 μm. CG chitosan/gelatin, PHA polydopamine coated-hydroxyapatite, PBT polydopamine coated-barium titanate, TNF-α tumor necrosis factor-α, IL-10 interleukin-10, VEGF vascular endothelial growth factor, BMP-2 bone morphogenetic protein 2, Runx2 Runt-related transcription factor 2

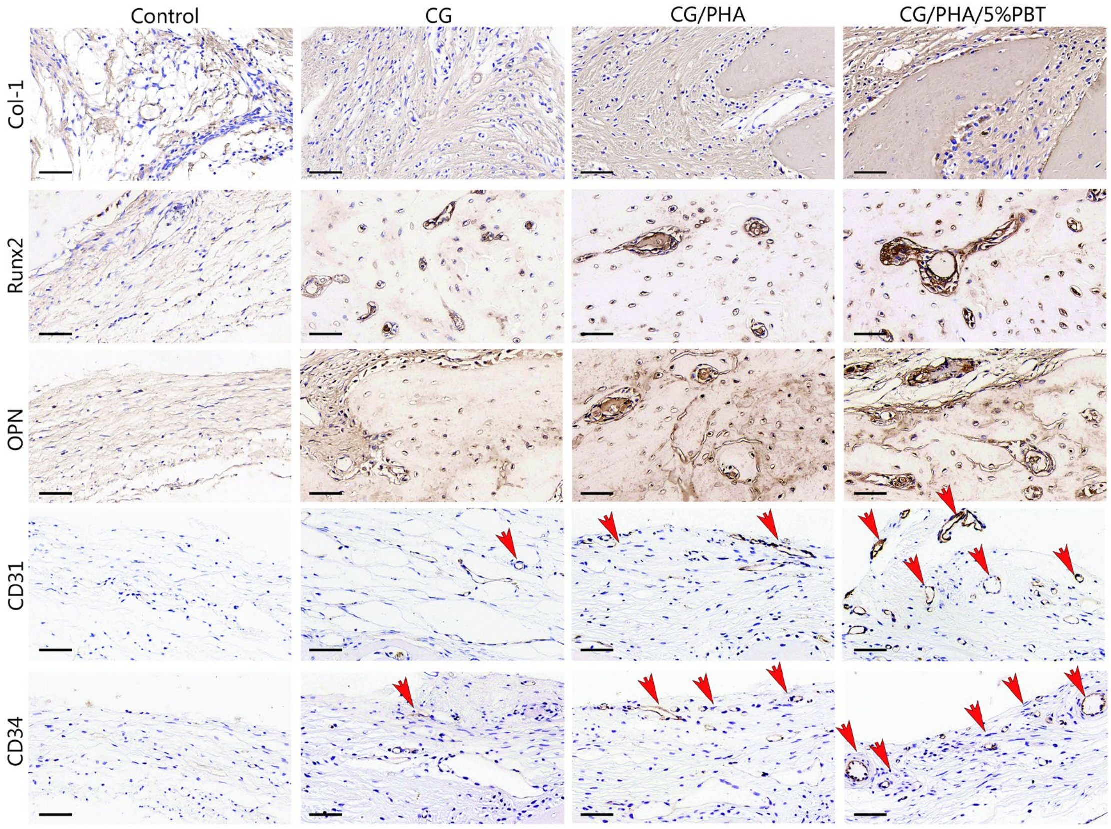

Fig. S8 Representative immunohistochemical staining images of Col-1, Runx2, OPN, CD31, and CD34 within the defect area 8 weeks after implantation.

The red arrows represent new blood vessels. Scale bar: 200 μm. CG chitosan/gelatin, PHA polydopamine coated-hydroxyapatite, PBT polydopamine coated-barium titanate, Col-1 collagen type I, Runx2 Runt-related transcription factor 2, OPN osteopontin, CD31 platelet endothelial cell adhesion molecule-1, CD34 hematopoietic progenitor cell antigen

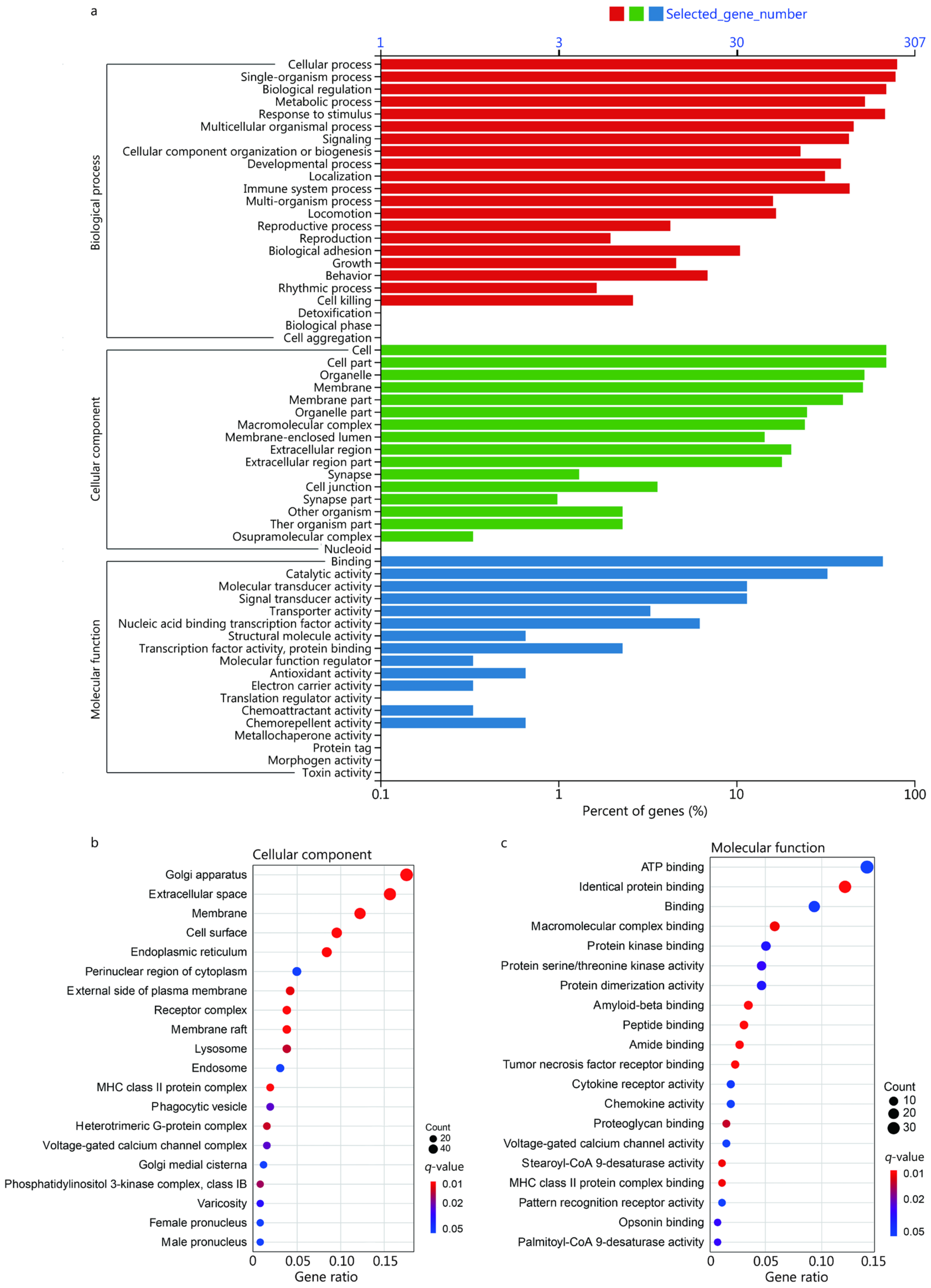

Fig. S9 Results of transcriptome sequencing of macrophage under the piezoelectric hydrogel stimulation.

a Gene ontology (GO) annotations of the assembled unigenes in macrophage under the piezoelectric hydrogel stimulation. **b** GO enrichment analysis of the 20 most differentially up-regulated and down-regulated cellular components. **c** GO enrichment analysis of the 20 most differentially up-regulated and down-regulated molecular functions

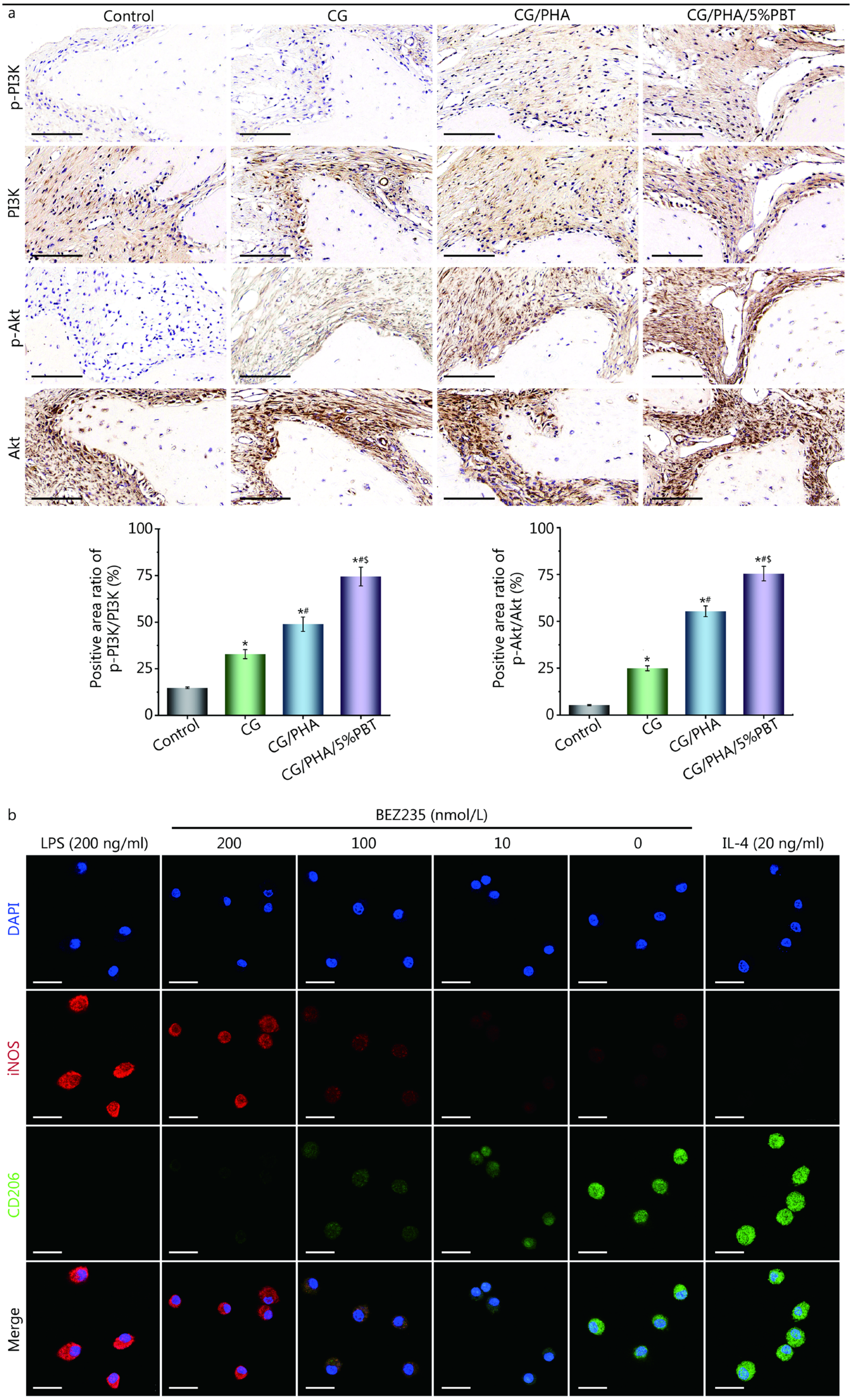

Fig. S10 The CG/PHA/5%PBT piezoelectric hydrogel regulates bone repair *via* the PI3K/Akt axis.

a Immunohistochemical images of p-PI3K, PI3K, p-Akt, and Akt within the injured sites after

implantation and the quantitative analysis of the immunohistochemical assay. Scale bar: 100 μm. **b** Representative immunofluorescence analysis of the inhibition test. Piezoelectric hydrogel can activate the PI3K/Akt signaling pathway. We verified the mechanism of piezoelectric hydrogel by inhibiting PI3K/Akt signaling pathway with inhibitors BEZ235. Scale bar: 20 μm. $^{*}P < 0.05$, compared with the control group; $^{\#}P < 0.05$, compared with the CG group; $^{\$}P < 0.05$, compared with the CG/PHA group, $n = 3$. CG chitosan/gelatin, PHA polydopamine coated-hydroxyapatite, PBT polydopamine coated-barium titanate, p-PI3K phosphorylated phosphoinositol 3 kinase, PI3K phosphoinositol 3 kinase, p-Akt phosphorylated seronine protein kinase, Akt serine/threonine protein kinase
